# Last glacial loess dynamics in the Southern Caucasus (NE-Armenia) and the phenomenon of missing loess deposition during MIS-2

**DOI:** 10.1038/s41598-022-17021-5

**Published:** 2022-08-02

**Authors:** Daniel Wolf, Johanna Lomax, Lilit Sahakyan, Hayk Hovakimyan, Jörn Profe, Philipp Schulte, Hans von Suchodoletz, Christiane Richter, Ulrich Hambach, Markus Fuchs, Dominik Faust

**Affiliations:** 1 Institute of Geography, Technische Universität Dresden, Helmholtzstr. 10, 01069 Dresden, Germany; 2 Department of Geography, Justus Liebig University Giessen, Senckenbergstr. 1, 35390 Giessen, Germany; 3 Institute of Geological Sciences, National Academy of Sciences of the Republic of Armenia, Baghramyan Ave. 24a, 0019 Yerevan, Armenia; 4 Department of Geography, RWTH Aachen University, Wüllnerstr. 5b, 52062 Aachen, Germany; 5 Institute of Geography, Leipzig University, Johannisallee 19a, 04103 Leipzig, Germany; 6 BayCEER & Chair of Geomorphology, University of Bayreuth, 95440 Bayreuth, Germany

**Keywords:** Climate change, Palaeoclimate, Climate-change ecology, Palaeoecology

## Abstract

The Marine Isotope Stage (MIS) 2 is considered the coldest, driest and stormiest period during the last Glacial-Interglacial cycle in large parts of Eurasia. This resulted from strongly decreased northern hemisphere temperature and related maximum extension of northern ice sheets that strongly reinforced large-scale circulation modes such as westerlies and East Asian Winter Monsoon driven by the Siberian High. Normally, this intensified circulation is reflected by maximum loess deposition in numerous loess regions spanning Europe and Asia. However, here we present a new loess record from the Caucasus region in NE-Armenia providing evidence in support of heavily reduced or even lacking loess formation during the MIS-2. Owing to implementations of comprehensible luminescence dating work and a provenance survey using rock magnetic and geochemical data, we are able to define distinct loess formation phases and to retrace sediment transport pathways. By comparing our results to other Eurasian palaeo-records, we unveil general atmospheric circulation modes that are most likely responsible for loess formation in the Southern Caucasus. Moreover, we try to test different scenarios to explain lacking loess formation during MIS-2. In line with other archive information, we suggest that loess formation was hampered by higher regional moisture conditions caused by a southward-shift of westerlies and renewed moisture absorption over the Black Sea. Our results show that modifications of MIS-2 circulation modes induced a very heterogeneous moisture distribution, particularly in the lower mid-latitudes of Eurasia producing a juxtaposition of very dry (morphodynamically active) and moderately dry (morphodynamically stable) areas.

## Introduction

Understanding mechanisms and causes of environmental change in terrestrial ecosystems occupies a key role with respect to past, present and future climate changes in Eurasia. However, reconstructing the history of such relations, in particular for the last glacial period, poses a substantial challenge all the more considering the scarceness of suitable terrestrial archives in many places. In recent decades, Loess–Palaeosol-Sequences (LPS) gained importance in providing high resolution palaeoenvironmental information on different regions of Eurasia. This is based firstly on the widespread distribution of loess deposits^1,2^, and secondly on the high level of detail provided by well-investigated loess records. Owing to remarkable progress in the field of age determination, litho-/pedostratigraphic features in LPS can be linked to millennial to centennial-scale climate variations^3,4^. This is exemplified by impacts of Dansgaard-Oeschger (DO) oscillations in the North Atlantic on climate and environmental changes in Western and Eastern Europe^3–8^. Likewise, regarding different Asian regions a teleconnection to North Atlantic patterns is widely accepted, e.g. based on the conformity of loess records between Europe and East Asia^6,9–11^. Hereby, position and strength of the westerly jet are assumed to be connecting elements that interacted with characteristics and location of Siberian High and Asian Monsoon as the main factors controlling rainfall variability and wind strength in Eastern Asia^10,12–14^. A similar interplay between westerlies and the Siberian High is considered significant for loess dynamics in Central Asia [^13,15,16^]. A frequent feature in all mentioned regions stretching from Western Europe towards Eastern Asia is the increase of dust flux and loess sedimentation rate during Marine Isotope Stage (MIS) 2^3,4,17–26^. In particular, loess sedimentation intensified during the Last Glacial Maximum (LGM) (26–19 ka) in line with the coldest part of the last glacial cycle as well as peaking global ice volume^27^. Accordingly, aside from the availability and proximity of dust sources as controlling parameter^28,29^, periods of enhanced loess sedimentation are often related to environmental conditions including strongly reduced precipitation and moisture availability, diminished vegetation cover, and higher wind speeds^23,30,31^.

In our study, we present a new loess record from the Southern Caucasus region where maximum last glacial loess sedimentation took place during MIS-4 and MIS-3, with just little evidence of loess sedimentation during MIS-2. It is discussed whether environmental conditions during MIS-2 may have disabled loess formation in the Caucasus region, in contrast to other loess regions in Europe and Asia. Therefore, the primary objective of this study is to characterize the main loess sources, and thus the main mode of loess formation in the Southern Caucasus region by conducting a sediment provenance study with a combined geochemical and petrophysical approach. Based on this provenance study, we evaluated different influencing factors based on a comparison with local to regional palaeoenvironmental archives as well as climate records of regional to supra-regional relevance. Finally, we placed our results on last glacial loess formation periods into the context of changing atmospheric circulation patterns over Eurasia.

## Study site

**Figure 1:**
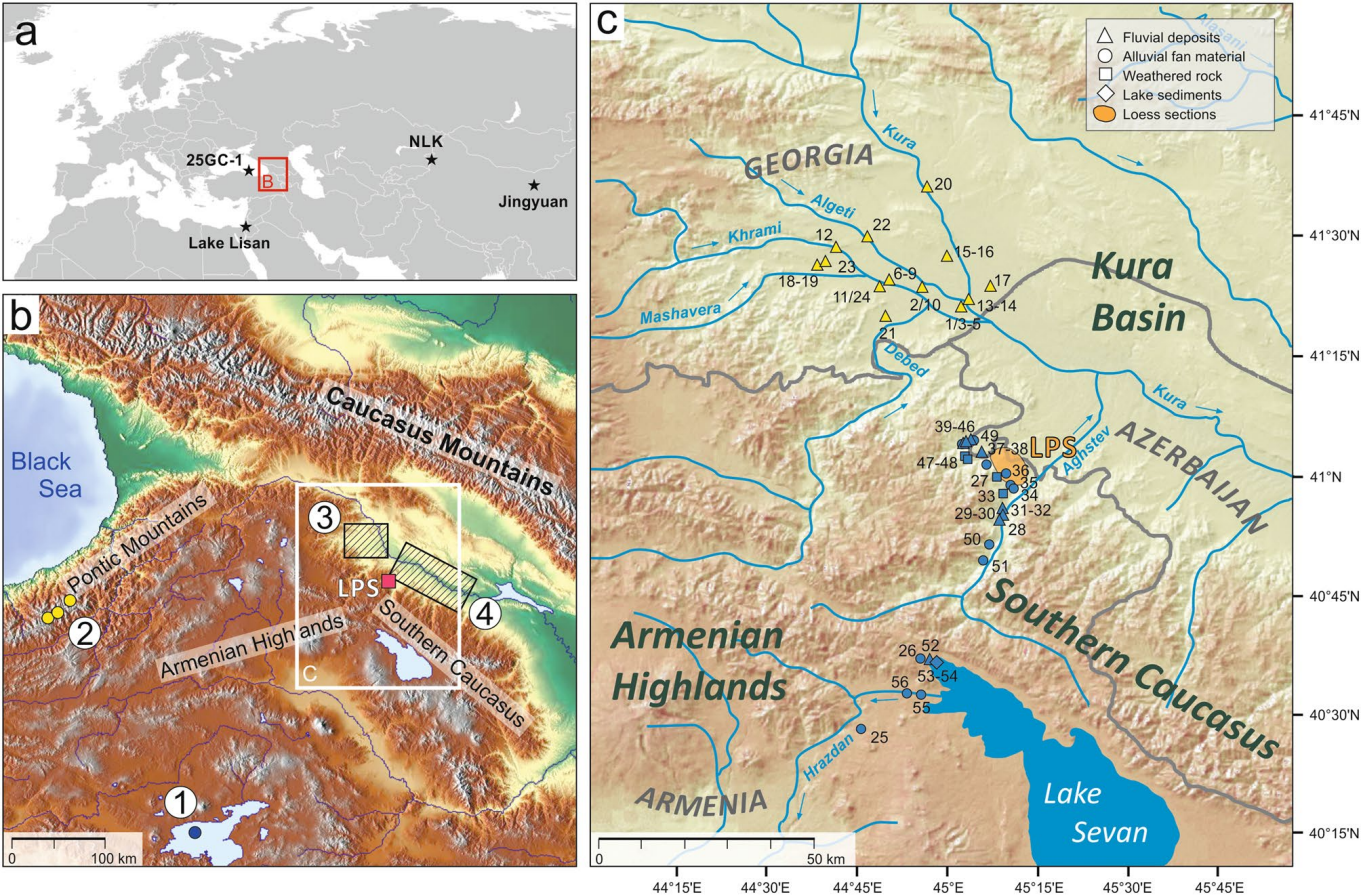
(**a**) Map showing the location of the study site in southern-central Eurasia with key palaeoenvironmental records (black stars) mentioned in the text: Black Sea record 25GC-1^82,93^, Lake Lisan record^80^, LPS Nilka (NLK)^22^, and LPS Jingyuan^64^. (**b**) Map of the Caucasus region. Studied LPS in NE-Armenia are indicated by a purple square. Further key palaeoenvironmental records are highlighted by numbers: (1) Lake Van (blue circle)^81^, (2) glacier record in the Pontic Mountains (yellow circles)^89^, (3) fluvial record of the western Kvemo Kartli Plain in Georgia (hatched area)^85,114,115^, (4) fluvial record from the middle Kura Valley (hatched area)^83^. (**c**) Map showing the sampling locations in the Kura Basin (yellow) and the Southern Caucasus and Armenian Highlands (blue) used for the provenance survey. Maps are based on (http://www.maps-for-free.com) and Global Multi-Resolution Topography Synthesis^116^.

The studied sections belong to a loess area in NE-Armenia, stretching along the northeastern foothills of the Armenian Highlands at the transition to the lowlands of the Kura Basin (Fig. 1). The area reveals a complex orographic configuration that likewise strongly modifies the influence of atmospheric circulation patterns. In general, the region lies at the interface between the temperate climate zone to the North and the subtropical zone to the South. In the North, the massif of the Greater Caucasus shelters the Kura Basin against direct impacts of cold air masses coming from arctic regions. Present-day climate conditions in the northern Armenian Highlands strongly depend on position and strength of westerly storm tracks as well as the Siberian High pressure system (SH)^32,33^. Back-trajectory analyses revealed three main modes of moisture transport to the Armenian Highlands: (i) with westerlies from the North Atlantic and the Mediterranean Sea (45%), (ii) via a southerly route from the Red Sea and Persian Gulf (25%), and (iii) along an easterly route crossing the Caspian Sea (30%)^33^. During winter, cold continental air masses may penetrate the area from eastern directions. However, in which way changes in position and strength of westerly storm tracks or the SH during the Pleistocene period affected the Armenian Highlands and the Kura Basin is largely unknown so far.

**Figure 2:**
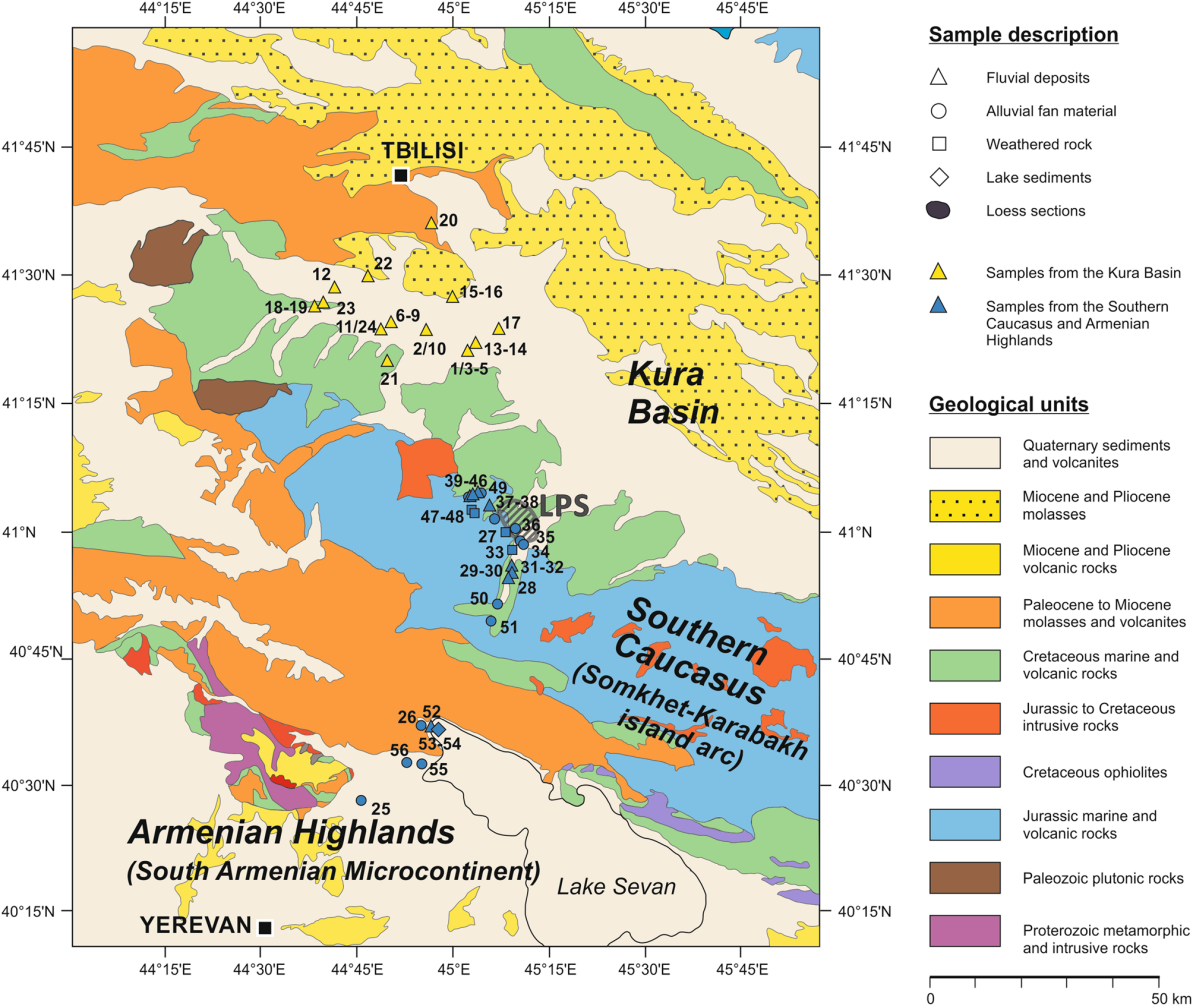
Map showing the sampling locations for the provenance survey with respect to geological units. The map was generated based on data from the Geological Map of Republic of Armenia 1:500.000^37^ and the Geological Map of the Caucasus 1:500.000^117^.

The immediate study area at the northeastern flanks of the Southern (Lesser) Caucasus shows mean annual rainfall of 450–550 mm and a mean annual temperature of 11 °C. It represents an area of pronounced gradients in terms of topography, temperature and precipitation that is finally reflected in a strong vertical vegetation zonation. Areas covered by loess are mainly used for agricultural purposes, while the potential natural vegetation would indicate a belt of temperate to subtropical forests merging into subalpine and alpine meadows at higher altitudes to the southwest, and steppes as well as semi-deserts in the Kura Basin to the northeast^34^. During glacial periods of the Pleistocene, semi-desert and shrub vegetation spread from the Kura lowlands up the slopes towards the today's loess distribution area^35,36^ indicating pronounced aridity linked to phases of loess deposition. The geological structure of the study area is characterized by a sequence of Jurassic, Cretaceous and Paleogene volcanic formations and volcaniclastic rocks belonging to the Southern Caucasus (Somkhet-Karabakh terrane) to the southwest (Fig. 2). The northeast is dominated by Cretaceous limestone, sandstone and volcaniclastic sedimentary rocks. The Kura Basin itself is characterized by Miocene and Pliocene molasses as well as Pliocene and Quaternary sediments (Fig. 2)^37,38^ (see Supplementary Information).

## Results

### Stratigraphy and chronology

**Figure 3:**
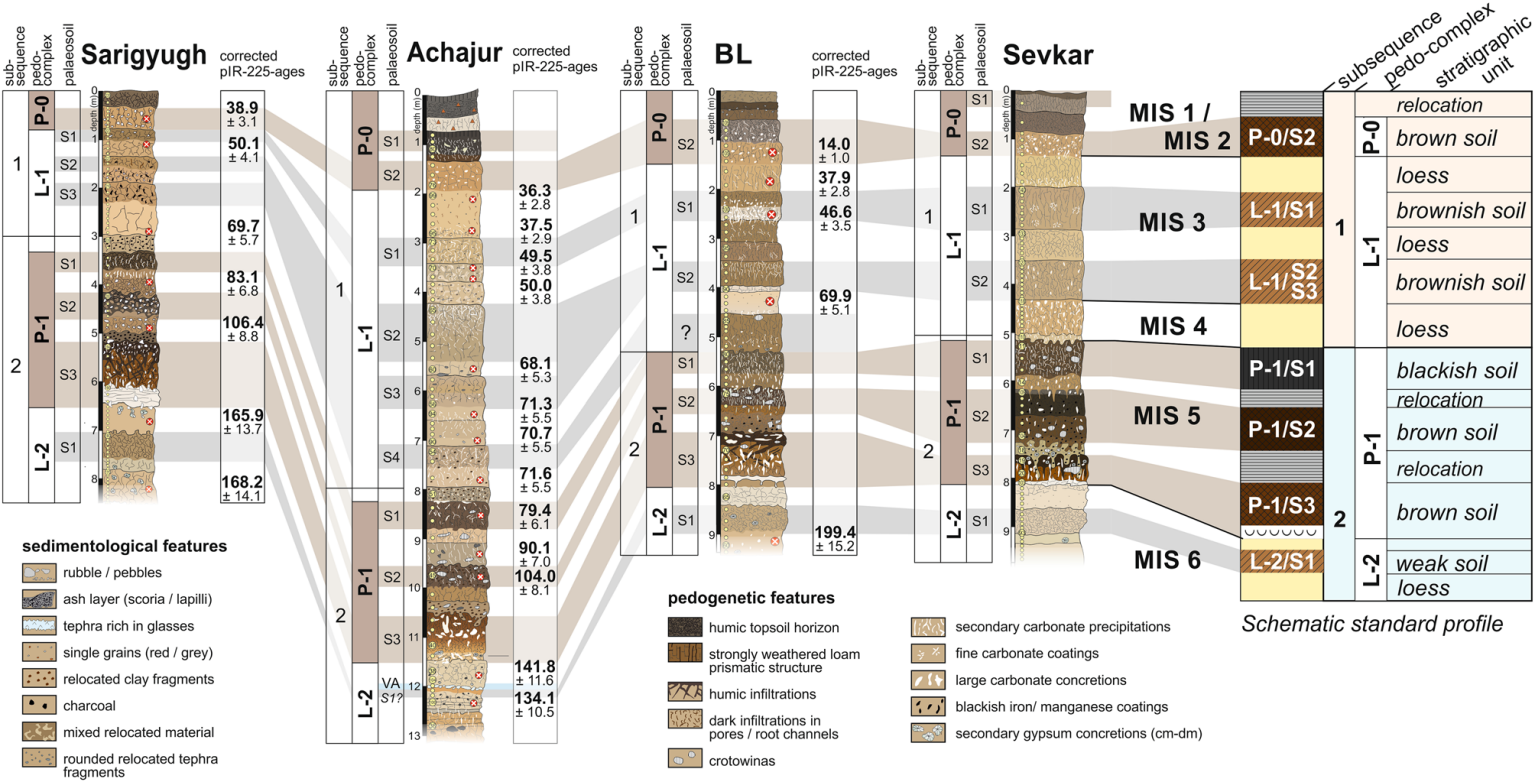
Stratigraphic correlation of the LPS Sarigyugh, Achajur, BL (borderline) and Sevkar in NE-Armenia for the last glacial-interglacial cycle. Palaeosoils belonging to pedocomplexes are indicated by brown colouring, and palaeosoils linked to glacial phases are marked by grey background. The correlation is strongly supported by the post IR-IRSL-225 chronology. On the right side, a simplified sketch offers a standard profile for the loess area in NE-Armenia with an overview on the most important palaeosoils and loess units together with a rough classification of the palaeosoils.

The stratigraphic sequence of the last glacial period appears to be very similar in all studied LPS sections (Fig. 3). Moreover, luminescence dating results show a precise match of depositional periods in all profiles.

Above the penultimate glacial loess (L-2), a three-parted pedocomplex is clearly visible in all four sections. The lowermost palaeosoil (P-1/S3) constitutes the most intense soil formation of this pedocomplex based on thickness, high clay and organic contents, polyedric aggregation and secondary calcium-carbonate enrichment in the subsoil (see Supplementary Fig. S1). The upper palaeosoil (P-1/S1) indicates a Chernozem-like soil type with pale black colors, a crumbly structure, and plenty of krotovinas. Based on luminescence dating in terms of fading corrected post-IR-IRSL (p-IRIR) ages^39^, we assign pedocomplex P-1 to MIS-5. The lower palaeosoil (P-1/S3) relates to the Eemian interglacial period (MIS-5e) as evidenced by pedogenic features and p-IRIR ages. P-1/S2 was formed between 106.4 ± 8.8 and 90.1 ± 7.0 ka (during MIS-5c), and P-1/S1 developed during MIS-5a in a period between 83.1 ± 6.8 and 71.6 ± 5.5 ka (Fig. 3 and Supplementary Table S1). After a phase of sediment relocation, loess deposition set in at about 71.6 ± 5.5 ka during MIS-4, a period of global drying and cooling. Instead of ongoing more or less continuous loess formation during the last glacial period, we rather found clear and brief phases of deposition during MIS-4 (71.6 ± 5.5 ka to 68.1 ± 5.3 ka) as well as MIS-3 (50.1 ± 4.1 to 46.6 ± 3.5, and 38.9 ± 3.1 to 36.3 ± 2.8 ka, Fig. 3 and Supplementary Table S1). These last glacial loess layers contain several intercalated palaeosoils, evidencing strongly reduced or even absent dust input with coeval pedogenesis. The partly varying number of palaeosoils between the sections reflects site-specific differences in sedimentation amounts and discontinuities in the sedimentation process. All sections are completed by a soil characterized by a reddish subsoil bearing plenty of secondary carbonate concretions and a blackish topsoil rich in organic carbon (P-0/S2). Since clay contents within that subsoil do not exceed 30% and soil colour as well as aggregation are much weaker compared to soils from other pedocomplexes, this soil resembles an interstadial palaeosoil rather than an interglacial one. There are different pIRIR ages from the lower part of this soil that indicate ages between 38.9 ± 3.1 ka (Sarigyugh section) and 36.3 ± 2.8 ka (Achajur section). Solely the BL section (Fig. 3) yielded a more recent age of 14.0 ± 1.0 ka, probably relating to a last pulse of loess sedimentation associated with the final stages of MIS-2. It is therefore possible that the subsoil of P-0/S2 was already formed during certain periods of MIS-2 and/or late MIS-3 instead of being a pure Holocene formation.

Apart from the single late glacial age, none of the sections provides indications of significant loess formation during MIS-2 including the LGM. However, in many places worldwide, the MIS-2 and especially the LGM relate to most intensive loess formation^1,20,40^. Lacking MIS-2 loess may be a result of post-depositional erosion processes. Therefore, we studied the road-cuts of each loess section along the longitudinal direction for more than 100 m for identifying hiatuses and erosion discordances (Supplementary Figs. S2–S4). Massive erosion events coincide with the penultimate glacial (Supplementary Fig. S2), the MIS-5 pedocomplex (P-1, Supplementary Fig. S4), and the middle to late Holocene period (Supplementary Figs. S2–S4), but we found no erosion discordances during the upper last glacial period. Instead, respective loess layers run uniformly and parallel with constant thickness, which rather contradicts serious erosion events. Consequently, we suggest that little or no loess was formed in NE-Armenia after MIS-3, which represents a considerable asynchrony with other northern hemispheric loess records. In order to approach the causes for this asynchrony, we applied a provenance study for defining the main source of loess. This raises the question of whether conditions at the loess source may have prevented loess formation during MIS-2.

### Provenance survey

The provenance survey was realized by means of XRF-measurements as well as the determination of rock magnetic patterns. We identified two potential loess source areas that are (i) weathered rocks and fine sediment-bearing fluvial and alluvial deposits in the Southern Caucasus and Armenian Highlands, and (ii) the Quaternary basin fill of the Kura Basin (Fig. 2). In addition to 33 samples from the LPS (Supplementary Fig. S1), we included 24 sediment samples from the Kura Basin (Supplementary Table S2) and 34 sediment samples from the Southern Caucasus and Armenian Highlands (Supplementary Table S3) into the provenance study.

**Figure 4:**
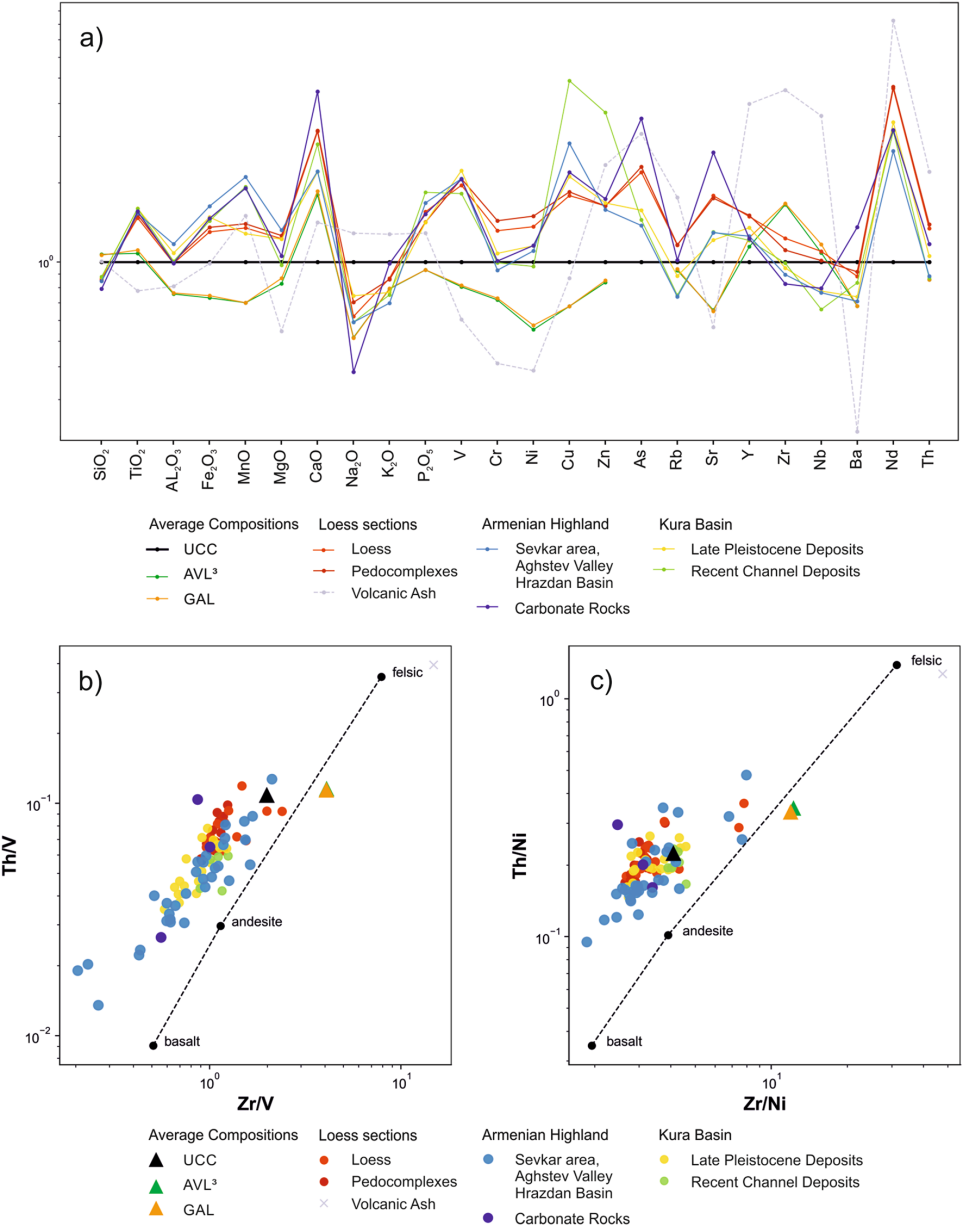
Geochemical information of loess and provenance samples. (**a**) UCC-normalized element patterns. The lines represents the mean values of the different lithological groups (*UCC *upper continental crust^41^, *AVL* average Loess composition^42^, *GAL *global average loess composition^42^). (**b,c**) Zr/metal vs. Th/metal ratios. LPS samples as well as samples from potential dust sources plot close to the UCC left from the compositional trend line showing igneous rock composition. Thus, there is no hint for sediment recycling induced zircon enrichment.

Loess in NE-Armenia reveals an elemental composition that differs from other known loess areas in Eurasia. In comparison with the Upper Continental Crust (UCC)^41^, the Average Loess Composition (AVL) and Global Average Loess Composition (GAL)^42^, both LPS and potential source areas show depletion in SiO_2_ and Zr whereas enrichment in TiO_2_, Fe_2_O_3_, MnO and compatible elements such as V, Cr, Ni, Cu (Fig. 4a). This suggests rather intermediate to mafic rocks as parent material. Such chemical composition is unusual for most European and Asian LPS, but in agreement with regional volcanology and geology^43–45^. Zr/metal vs. Th/metal ratios depict that the LPS and potential dust source samples plot close to the UCC left from the compositional trend line indicating igneous rock composition^42^ in Fig. 4b,c. Thus, sediment recycling induced zircon enrichment seems unlikely while short-range transport of fresh material seems likely. Finally, there is just little geochemical evidence of one particularly recognizable loess source, since most of our three sample groups plot closely together in all diagrams (Fig. 4a–c). While the tephra layer is clearly visible within the plotted element profiles (Fig. 6, volcanic ash in 10 m depth), there is no hint for dust source changes along the profile. Using Cl as tephra or dust provenance proxy^46^ is complicated by the location of the studied LPS in a semi-arid region as formation of evaporites or remnants of marine salt deposits^47^ cannot be ruled out as causes for local maxima in the profiles of Cl, S and Na (Fig. 6). Instead, compatible elements such as Ni and Cr together with conservative element ratios such as Zr/Ti and Al/Ti may help to identify tephras and dust provenance changes along the profile^29,46,48^. The major tephra layer suggests rather a rhyolithic character with minima in compatible elements and maximum in Zr (Fig. 6). Accordingly, three more cryptotephras might be hypothesized at depths of 26 m, 16.6 m and 11 m. However, apart from these hypothesized cryptotephras, the element profiles follow more straight lines rather than interpretable fluctuations. The coarse spatial sampling resolution along the profile may prevent possible fluctuations from being sufficiently recognized in XRF data. Consequently, element data renders conclusion of one major dust source nearly impossible.

**Figure 5:**
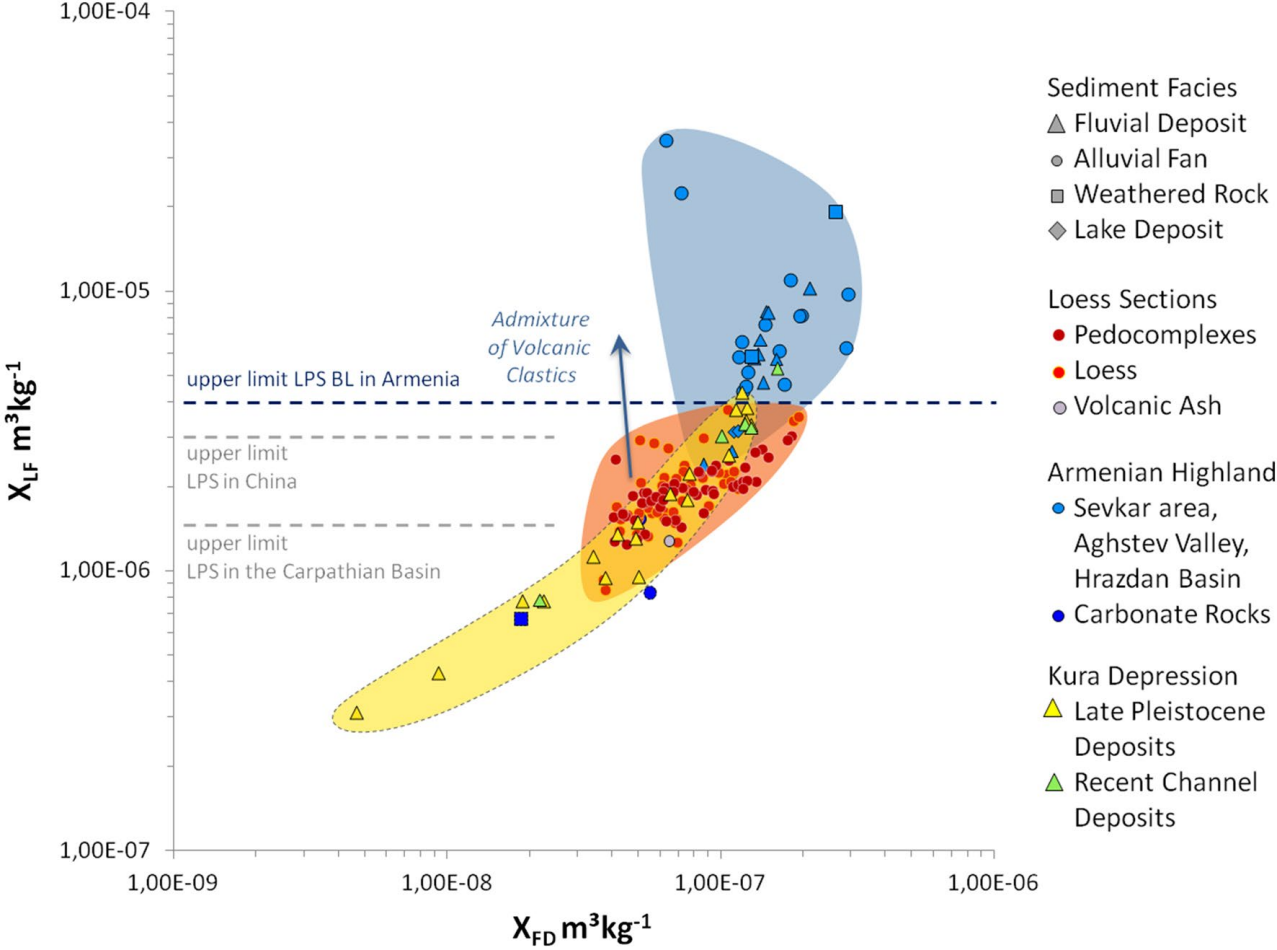
Low field magnetic susceptibility χ_LF Hz_ (in m^3^ kg^−1^) vs. frequency dependent susceptibility (χ_fd_ = χ_300 Hz_ − χ_3000 Hz_ in m^3^ kg^−1^). Samples from volcanogenic areas of the Armenian Highlands (blue) plot in the highest value ranges reflecting high contributions of magnetic ores. Samples from the mostly fluvial sediment infill of the Kura Basin (yellow and green) plot in lower value ranges, probably reflecting the admixture of eroded limestone (diamagnetic minerals). Samples from the LPS BL (red) plot in the same lower range as the Kura Basin samples, while higher χ_LF_ values within the loess indicate a higher admixture of clastic volcanic material. Horizontal dashed lines indicate the upper limit of χ_LF_ in LPS of NE-Armenia, China^50^ and the Carpathian Basin^49^. Most recent channel deposits from the Kura Basin (green) plot in the same highly magnetic ranges as the volcanogenic samples from the Armenian Highlands, indicating that these current fluvial deposits are mainly fed by volcanic sources from the upper catchment areas and not by the surrounding Kura Basin infill.

**Figure 6:**
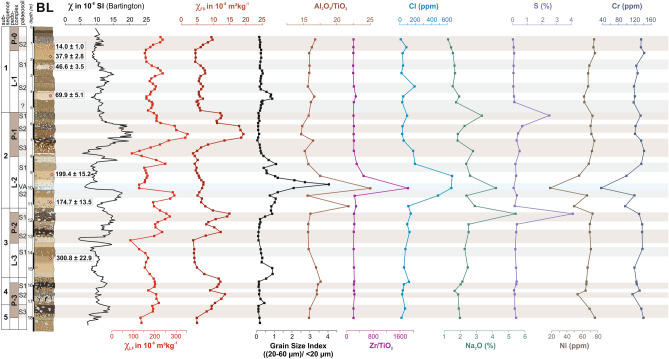
Depth variations of magnetic susceptibility (field measurements in SI units), low-frequency magnetic susceptibility (χ_lf_), frequency.dependant magnetic susceptibility (χ_fd_), Grain Size Index (GSI, after^18^), ratios of Al_2_O_3_/TiO_2_ and Zr/TiO_2_, Cl in ppm, Na_2_O in %, S in %, and Ni and Cr in ppm, resulting from XRF and rock magnetic measurements in the LPS BL. Please note that sampling density between rock magnetic measurements/grain-size measurements and XRF measurements is varying.

In contrast, rock magnetic patterns clearly indicate that most of the loess deposits should originate from the Kura Basin. Compared with loess samples from other regions, all LPS samples from Armenia show relatively high low field magnetic susceptibility (χ_lf_) values of up to 400 × 10^–8^ m^3^ kg^−1^ (Fig. 5) that cannot merely be related to magnetic particle formation during weathering and pedogenesis. In contrast, χ_lf_ of soils formed in loess in the Carpathian Basin reveal maximum values of only 150 × 10^–8^ m^3^ kg^−1^^49^, while χ_lf_ in China reaches somewhat higher values of up to 300 × 10^–8^ m^3^ kg^−1^ related to stronger pedogenesis due to monsoonal effects (Fig. 5)^50^. Likewise the potential source areas of the Armenian loess exhibit high χ_lf_ values suggesting that the parent material of the Armenian loess is not comparable to that of loess common in SE Europe or China, but is strongly affected by weathered volcanogenic minerals as it is known from the vast loess areas of Argentina and southern Brazil^51,52^. The high magnetic susceptibility (MS) in LPS of NE-Armenia may relate to high proportions of metallic ores within volcanic glasses that attain background levels of up to 20% within unaltered loess^53^. In addition to ultrafine superparamagnetic (SP) particles released from volcanic rocks and glasses, another phenomenon contributing to high χ_lf_ values is the surface oxidation of relatively large detrital and/or magmatic magnetic grains forming a crust of SP-domains^54–56^. These processes are possibly responsible for the Armenian Highland samples occupy the highest χ_lf_ value range (Fig. 5). On the contrary, samples from the Kura Basin show lower χ_lf_ value ranges, reflecting the admixture of diamagnetic mineral fractions from eroded Cretaceous limestone (Figs. 2 and 5). Similarly low χ_lf_ values are derived from weathered carbonate rocks in the Armenian Highlands (Fig. 5, dark blue circles) and in pedogenic carbonate crusts within the LPS (Fig. 6, e.g. P-1/S3 and P-2/S2). The LPS samples largely plot within the range of the Kura Basin samples, which is lower than that of the Armenian Highland samples. Loess may experience an MS increase in case of weathering and pedogenesis (magnetic enhancement accompanied by increasing χ_fd_) or due to admixed volcanic clastics (Fig. 5)^56^, while post-sedimentary MS depletion is highly improbable in case of the LPS in NE-Armenia since no signs of requisite water-saturated conditions^57^ were identified in the sections. Increasing MS in line with pedogenesis is, e.g., visible in the supposed Eemian soil P-1/S3 (Fig. 6), although the rise of 300 × 10^–8^ m^3^ kg^−1^ appears to be too large for mere pedogenic origin. Instead, we assume a higher contribution of volcanogenic material for the last interglacial period. Likewise, the palaeosoil assumedly linked to MIS-5c (P-1/S2, Fig. 6) revealed maximum MS-values. However, the respective parent material deposited around 106.4 ± 8.8 ka (Fig. 3) shows even higher MS-values that may suggest a changed dust source during that period. Referring to the Al/Ti ratio that is regarded as an indication of grain-size and provenance changes^29^, stronger deviations from the Grain Size Index (GSI) curve solely appear in the range of the upper Eemian soil P-1/S3 and the loessic material at the base of the L-2 complex (Fig. 6, depth of 7 and 11 m) that may point to a provenance change and/or higher tephritic input. Since no clear indications arise from elemental analyses (Fig. 6) we can just assume that these patterns reflect a strong dust input from the Armenian Highlands probably due to stronger west winds as is the case today^33^. Similar but weaker MS-increases are linked to the palaeosoils of pedocomplexes P-3 and P-2, while the youngest pedocomplex P-0/S2 shows just very weak MS-enrichment.

## Discussion

**Figure 7:**
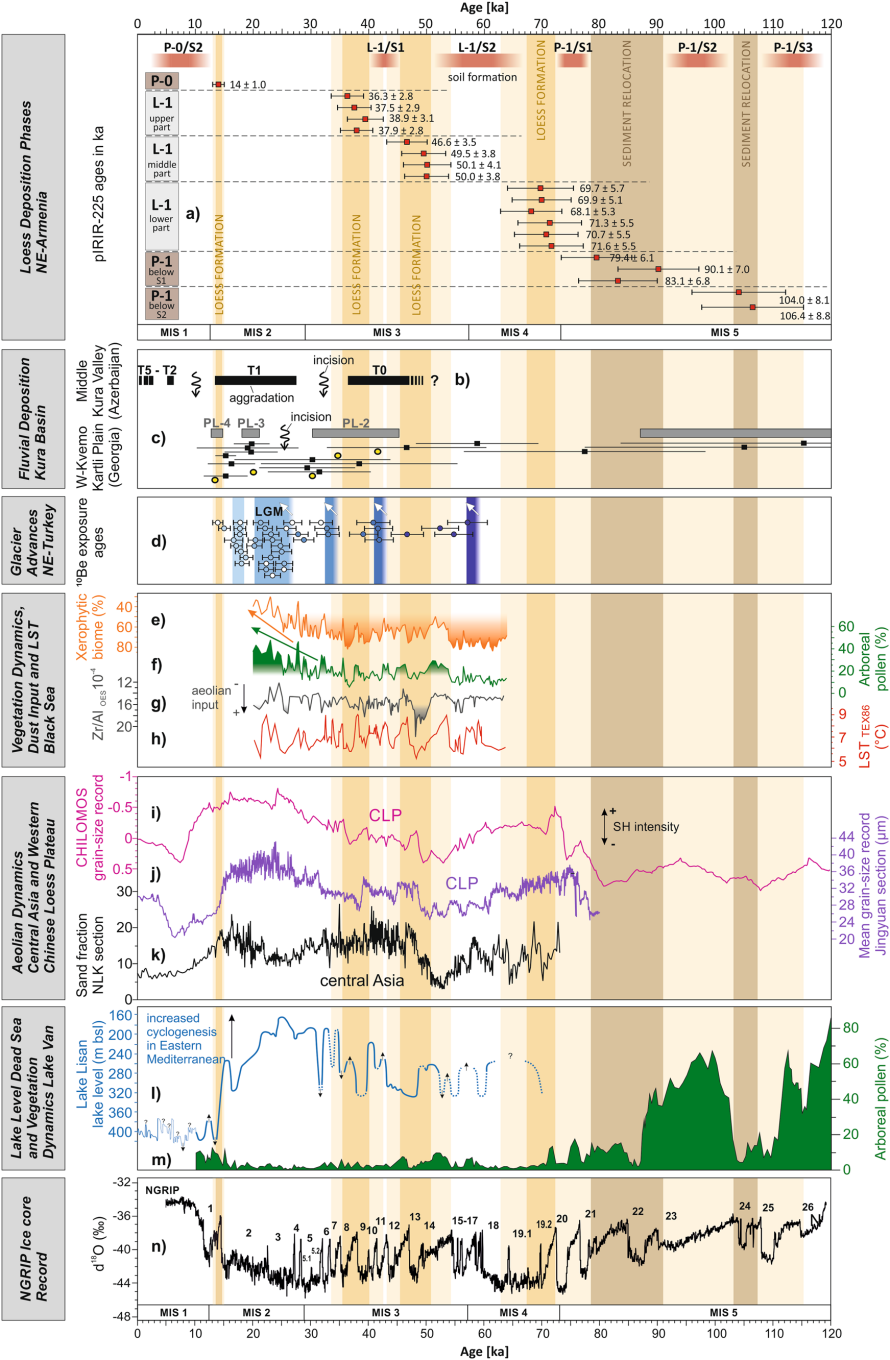
Main loess deposition (vertical ochre bars) and sediment relocation (vertical brown bars) phases in NE-Armenia during the last 120 kyrs compared with other palaeoenvironmental records of regional to supra-regional significance. (**a**) Age model of the loess record in NE-Armenia based on post-IR-IRSL-225 dating results together with indication of soil formation periods. Mean ages are shown together with relative errors (for details, see Supplementary Table S2). (**b**) Fluvial aggradation and incision periods in the middle Kura Valley, Azerbaijan. T0–T5 indicate different fluvial terrace levels. Continuation of aggradation before 47 ka is not documented but likely based on stratigraphic information beyond the radiocarbon dating limit^83^. (**c**) Fluvial aggradation and incision periods from the western Kvemo Kartli Plain, Georgia. PL-2–PL-4 indicate different fluvial terrace levels. Sedimentation ages (black squares = OSL dating; yellow circles = ^14^C dating) with relative errors are shown^85^. (**d**) Glacier advances in the Pontic Mountains, NE-Turkey, based on ^10^Beryllium exposure ages. White arrows indicate times of maximum ice advance^89^. (**e–h**) Proxy information from coring 25GC-1 in the SE-Black Sea including: pollen percentages linked to xerophytic biome (**e**), and percentages of arboreal pollen (**f**)^93^, as well as Zr/Al-ratios indicating aeolian input (**g**)^82^ and GDGT-lipide (TEX_86_) based mean annual lake surface temperatures (**h**)^94^. Green and orange arrows mark an increasing regional humidity trend during the LGM. (**i**) Stacked normalized grain-size record from northern China (CHILOMOS), with negative value ranges indicating grain-size coarsening^65^. (**j**) Mean grain-size record from the LPS Jingyuan in the western Chinese loess plateau^64^. (**k**) Variation of the sand-fraction (> 52 µm in %) of the Nilka (NLK) loess section in central Asia^22^. (**l**) Lake level curve of Lake Lisan in the Dead Sea Valley. Note that the scale is in m below sea level. Higher lake levels are interpreted as linked with higher moisture supply due to increased cyclogenesis in the Eastern Mediterranean Sea^80^. (**m**) Percentage of arboreal pollen in the Lake Van record in eastern Anatolia, E-Turkey^81^. (**n**) δ^18^O record of the NGRIP ice core with numbers referring to Greenland stadials^60^.

The loess record in NE-Armenia highlights alternations between intense and strongly reduced loess deposition, the latter with accompanying soil formation testifying changing palaeoenvironmental conditions. Based on the current state of knowledge, these changes may relate to position and strength of westerly storm tracks and the SH as the dominant atmospheric circulation patterns in the Caucasus region. The precision of the chronological resolution of our loess record precludes a correlation of loess deposition phases with DO-cycles as dominant signals of past climate variability over the North Atlantic region^58,59^. However, loess deposition phases may include some of the most notable North Atlantic cold events^60^ linked to very low Sea Surface Temperatures (SSTs)^61^ that are Greenland Stadials (GS) 19, 13 and 9 (or 8) (Fig. 7n). The latter two likewise include Heinrich events 5 and 4. In that case, loess deposition corresponds to the coldest stages at the end of the glacial Bond-cycles^62^ at least during MIS-4 and lower to middle MIS-3. There are, however, a number of other Greenland Stadials and Heinrich events especially during MIS-2 that are not covered by loess deposition in our record. This begs the question whether last glacial North Atlantic climate dynamics were at all mirrored by similar climatic and environmental conditions in the Caucasus region.

In order to contextualize last glacial environmental dynamics in the Caucasus area, we first consider the interplay of different large-scale atmospheric circulation patterns affecting Eurasia. Moisture availability in Caucasia and adjacent regions is strongly influenced by the westerly jet and associated storm track positions, the SH with related cold and dry continental air masses, and moisture-bearing Mediterranean cyclonic frontal systems^22,33^. With respect to loess formation, it has been argued that loess deposition in higher-latitude Europe and mid-latitude Asia referred to interconnected large-scale atmospheric circulation patterns as evidenced by synchronous LPS build-up^6,9^. For instance, periods of strengthened East Asian Winter Monsoon (EAWM), reflected by grain-size coarsening in loess sections of the Chinese Loess Plateau (CLP), correlate with North Atlantic climate events^63–65^. Such North Atlantic climate events are likewise demonstrated in LPS in Europe^7,8,66–68^. The EAWM is driven by the SH^10^ that, in turn, gains strength from cold air supply from Arctic regions such as the Nordic and Barents Seas^65^. Thus, EAWM strength closely relates to ice volume and temperature of the northern high latitudes^10,65^. At the same time, this northern hemisphere temperature decrease is connected with increasing meridional temperature gradients, and thus with stronger westerly winds in the mid-latitudes^11^. Similar to the CLP, increased dust accumulation in line with a strengthened SH is also reported for central Asia^15,69^, which may be a further indication of common Eurasian atmospheric forcing patterns related to LPS build-up^16^.

The first phase of Weichselian loess deposition in NE-Armenia between 71.6 ± 5.5 and 68.1 ± 5.3 ka (Fig. 7a) coincides with the early MIS-4 that was characterized by lowest northern hemisphere summer insolation^70^. This cold phase of large-scale regional dryness^71^ was accompanied by massive loess deposition in large parts of Eurasia^8,15,19,72–75^ as well as intensified aeolian dynamics on the CLP (Fig. 7i,j)^65^. A global sea-level minimum and a strong peak in northern hemisphere ice-volume^76^ are consistent with intensified westerly winds and a strengthened SH. Apparently, these dynamics have caused a phase of increased landscape dynamics in the Caucasus area as shown by loess formation in NE-Armenia and dust accumulation in the Armenian Highlands^77^. From the next two loess deposition periods in NE-Armenia (50.1 ± 4.1 to 46.6 ± 3.5, and 38.9 ± 3.1 to 36.3 ± 2.8 ka, Fig. 3) especially the earlier one correlates with a strong increase of SH intensity as recorded on the CLP (Fig. 7i,j)^64,65,78^, but also with a North Atlantic cold period linked to GS-13 (Fig. 7n). A similar phase of higher wind strengths (~ 47 to 39 ka) was found in the Tien Shan Mountains^16^, and also the Carpathian and the lower Danube Basins in SE-Europe display both periods in form of loess deposition^74,79^. Moreover, these periods may potentially coincide with Heinrich events 5 and 4 that were also detected in the form of extreme drought episodes in the Eastern Mediterranean (Fig. 7l)^80^. Consequently, storms released from the westerly jet as well as strong surface winds linked to the SH may have initiated loess formation in the Southern Caucasus. Considering general circulation pathways from south-western directions^33^, severely cooled Eastern Mediterranean SSTs and related low atmospheric moisture absorption may have additionally caused strong aridity in the down-wind direction (Fig. 7l).

We are currently not able to differentiate between westerly jet and SH as main factors for dust storm formation in the Kura Basin. However, the fact that the frequency of loess deposition in NE-Armenia is more similar to large oscillations of grain-size proxies from central Asia and the CLP (Fig. 7k,j) than to Dansgaard Oeschger cycles (Fig. 7n) suggests that the SH might have been the more important trigger for generating dust storms in the Kura Basin as the main loess source during glacial periods. Since our provenance analyses point to northern to eastern winds during loess formation in NE-Armenia, this might be considered as a further indication for a stronger influence of the SH.

Loess deposition in NE-Armenia is rarely continuous, and is seemingly enabled through certain environmental conditions. Based on extensive gastropod analyses on the LPS in NE-Armenia^36^, we found that strong loess deposition in our study area was only possible under semiarid to arid climate conditions and thus, exaggerated aridisation in the Kura Basin (Supplementary Figs. S1 and S6). The increase in gastropod species typically corresponding to xerophilic communities (e.g. *K. crenimargo*, *G. interrupta*, *P.* aff. *poltavica*, see Supplementary Fig. S6) indicates that these communities lived in summer-warm semideserts such as shrub steppes. Since loess layers did not contain cold-adapted gastropod species, we assume that loess deposition was linked to drought, rather than to cold temperatures^36^. The periods in between strong loess deposition were characterized by more stable conditions and pedogenesis (Figs. 3 and 7a) as evidenced by strongly weathered palaeosoils (Supplementary Fig. S1), pointing to higher regional humidity (see also peaks of arboreal pollen in Fig. 7m)^81^ and reduced dust input. Based on our gastropod record, pedocomplexes are represented by species typical for high-grass and forest steppes (e.g. V*. pulchella, C. tridens, V. pygmaea*) demonstrating an average annual precipitation of above 300 mm (Supplementary Figs. S1 and S6). Higher humidity during the same periods was likewise reported for the Black Sea region and explained by retreated northern ice-sheets and a northward shift of the atmospheric polar front in line with maximum summer insolation^82^. This could finally have created the opportunity for increased precipitation in the Eastern Mediterranean and Caucasus area at the end of MIS-4 and during the middle MIS-3 related to approaching Mediterranean winter storm tracks^71^.

For the period after 36 ka, the density of proxy data coming from our loess record decreases significantly, thus impeding clear statements on palaeoenvironmental conditions. Based on our findings that suggest lacking loess deposits for MIS-2 and final MIS-3, we must assume that either loess formation strongly decreased, or formerly deposited loess strata were later removed. In the latter case, respective loess strata should have been cleared out during the Holocene or final stages of the last glacial epoch. However, we found no indications for serious erosion events such as erosion discordances, linear erosion features or colluvial deposits (Supplementary Figs. S2–S4). Moreover, stratigraphic and analytical findings show no evidence of material changes or unconformities within the uppermost part of the sections (e.g. between 1 and 2 m depth in BL section, Supplementary Fig. S1). With respect to lacking or strongly reduced loess formation, there are two conceivable scenarios. One scenario is that there was not enough dust for loess formation. Reasons might be severely restricted deflation processes due to reduced silt-supply, too low wind speeds or increased surface protection in the provenance area because of higher moisture availability. Another scenario is that there was enough dust, but no favourable conditions for trapping the dust. This could have been caused by missing vegetation cover due to increased dryness or by changed wind directions that hindered fallout in the study area. Both scenarios would be associated with a more or less stable or at least slowly accreting surface at our loess site, although very dry conditions should be rather linked to surface erosion instead of stability. While the second scenario, which includes utmost aridity, might have prevented soil forming processes, the first scenario should have led to soil formation within the loess record. We found no proof of a palaeosoil precisely attributable to the late glacial period, but in accordance with dating evidences and the special character of the suspected Holocene soil, it is however possible that its subsoil started to form already in a period before the Holocene, while there are strong indications that at least the topsoil is of Holocene age^36^. This in turn would mean an extreme reduction of loess formation during MIS-2 and a strong evidence of higher moisture availability in the study area.

At this point we must conclude that the missing or heavily reduced loess formation in NE-Armenia after 36 ka could be linked to different scenarios with greatly differing implications for palaeoenvironmental interpretation. With respect to the limited explanatory power of the LPS itself during this period, we realized a comprehensive review of various palaeoenvironmental and palaeoclimatic records from the wider region in order to provide the best possible approach for reconstructing late glacial loess dynamics in the Southern Caucasus.

Our provenance analyses identified the Kura Basin as potentially major source for loess deposits in NE-Armenia. Thus, as a first step we compared loess dynamics (Fig. 7a) with last glacial fluvial sedimentation patterns in the Kura Basin (Fig. 7b,c). A fluvial study from the middle Kura Valley^83^ in Azerbaijan, carried out very close to our loess sections (Figs. 1 and 7b), provides a very precise radiocarbon-based chronology. Accordingly, fluvial aggradation took place during middle MIS-3 and most probably also before^83^, but is suggested to have ceased suddenly around 35.3 ± 1.2 ka cal. BP. It was then followed by a period of serious river incision of up to 60 m before smaller-scale fluvial aggradation resumed for most part of MIS-2 (Fig. 7b). This termination of fluvial aggradation strongly agrees with the end of loess deposition in our record. Similarly, studies carried out in the western Kvemo Kartli Plain in Georgia and NE-Armenia (Figs. 1 and 7c)^84,85^ revealed the continuation of fluvial sedimentation of overbank floodplain sediments a bit longer until the end of MIS-3 at ca. 30 ka, followed by strong river incision of several decametres. Subsequently, fluvial aggradation with a much lesser extent continued during later stages of MIS-2. As a general relation, the transition from fluvial aggradation towards river incision should be accompanied by higher runoff and/or lower sediment supply, e.g., due to increasing rainfall and/or the development of a protecting vegetation cover in areas without permafrost^86,87^. Higher moisture availability in the Kura Basin and its adjacent mountain ranges should have led to higher runoff with a higher river transport capacity, and thus the initiation of streambed erosion. After deep river incision into the basin fill of the Kura Valley, the abandoned former floodplain surface was subsequently covered by vegetation or at least by biological soil crusts^88^. Finally, this should have stopped loess deflation from this surface, and the aeolian system may therefore have switched from a transport-limited into a supply-limited state. Information on glacier advances based on ^10^Beryllium exposure ages in the Pontic Mountains in NE-Turkey^89^ strongly support this scenario. Thereafter, glacier advances during the last 60 ka were anti-correlated with periods of loess deposition in NE-Armenia (Fig. 7d). Cold temperatures and especially increasing moisture availability play a key role for glacier growth in semi-arid regions^90–92^. While warm season temperatures control the glacier melting during retreat phases, the build-up of glaciers strongly depends on cold season snow precipitation. From this perspective, phases of glacier advances in the Pontic Mountains point to higher winter precipitation, while phases of loess deposition in the Armenian Highlands point to stronger aridity (Fig. 7a,d). A sediment record from the SE-Black Sea^93^ seems to suggest higher proportions of xerophytic vegetation and minima in arboreal pollen percentages contemporaneous with loess deposition (Fig. 7e,f). Even if this temporal correspondence cannot be proven without doubts, the strongly increased aeolian input between 47 and 50 ka^82^ together with lowered Black Sea surface temperatures^94^ (Fig. 7g,h) may be a further hint for regionally consistent environmental dynamics during MIS-3.

Similar relationships between loess deposition (glacier advances) and stronger aridity (higher moisture availability) have been documented for loess deposits in the nowadays semi-arid interior of Iberia^8^, or in semi-arid basins in central Asia with direct connection to the Tian Shan Mountains^16^. Therefore, we suggest that loess formation in the mid latitude Mediterranean belt and eastern adjoining semi-arid regions is mostly sensitive to higher aridity rather than to lower temperatures^1^. The complete absence of periglacial features within the loess sections together with the fact that mean July temperature in the region of the NE-Armenian LPS never fell below 10 °C during the last glacial period^36^, further support this hypothesis.

The surprising lack of loess formation in NE-Armenia during MIS-2 requires a closer examination of regional palaeoenvironmental patterns. Ongoing fluvial aggradation in the Kura Basin during most or at least parts of MIS-2 (Fig. 7b,c)^83,85^ may characterize the aeolian system as generally not being supply-limited, although strong entrenchment and the subsequently reduced width of the new Kura floodplain may have strongly reduced deflation processes. The Black Sea record does not indicate a serious temperature reduction until about 20 ka^94^ (Fig. 7h). Moreover, steadily increasing arboreal vegetation (Fig. 7f) and strongly reduced xerophytic vegetation^93^ (Fig. 7g) in the surroundings of the south-eastern shores of the Black Sea indicate a higher humidity during the LGM compared to MIS-3. Assuming the dominance of western winds over the Black Sea and Eastern Mediterranean Sea, moderate SSTs may have supported continuous atmospheric moisture flux towards downwind directions even during the LGM. This is further supported by glacier growth in the Pontic Mountains all over the LGM^89^ (Fig. 7d). Given a temperature decrease of about 10 °C during the LGM, calculations based on glacier modelling from SW-Turkey suggest that precipitation was twice as high as today^95^. Finally, even Lake Ohrid in Albania indicates a continuous increase of arboreal vegetation from an absolute minimum around 39 ka until a maximum during the Holocene^96^. All these records demonstrate a higher regional moisture availability during the LGM, that strongly contrasts with archive information from more continental Central and Eastern Europe and the western and northern shores of the Black Sea^66,79,97^.

After about 34 ka and especially during the LGM, the synchronism of loess sedimentation in Eurasia was largely replaced by different climatic responses in different regions^10,16,24^. The lowest global sea level, the most extended northern ice-sheets, and assumedly the strongest EAWM activity^15,64,65^ (Fig. 7i) were accompanied by partly strong differences of hydrological balance and temperature regime in Eurasia^66^. One of the main reasons for this is seen in the blocking effect of a high-pressure area over the increasing Fennoscandian ice-sheet that strongly modified the Rossby-wave curvature^98^ leading to a southward shift of the atmospheric polar front and west wind tracks over Southern and Southwestern Europe. This was partly accompanied by prevailing transport of air masses from south to north, bringing moisture from the Mediterranean Sea towards the European continent^92,98^. Recent modelling studies suggest an increase of southerly and even easterly weather types caused by anticyclone formation over the Fennoscandian ice-sheet during the LGM^23,99,100^. However, a general southward shift of westerly storm tracks from a northwest-to-southeast direction over Europe towards a west-to-east direction over the Mediterranean Sea is widely accepted for the LGM^101^. These mechanisms introduced a situation to Europe that was characterised by the coexistence of areas with serious environmental dryness, and neighbouring areas of reduced dryness or even more humid conditions (higher moisture availability)^46,102^. For example, early to middle Pleniglacial loess strata in central Europe characterized by several interposed weathering phases experienced a strong increase of accumulation rates after 34 ka, resulting in the formation of loess with much lower clay and organic carbon contents^6,7^. Likewise, also the Carpathian Basin was dominated by ever colder climate conditions, since the penetration of Atlantic air masses was blocked by the Fennoscandian anticyclon. This could have led to stronger physical weathering in the framing mountains and thus, to higher sediment supply and loess accumulation rates^4,79^. On the other hand, the pattern was different for areas under the influence of the southward shifted westerlies. For instance, the Dead Sea Basin was affected by strong cyclogenesis during the LGM that led to maximal moisture transfer from the Eastern Mediterranean Sea and thus, to maximum lake levels of Lake Lisan (Fig. 7l)^80^. Likewise, also Lake Ohrid and the SE-Black Sea region (Fig. 7e,f) recorded higher humidity during the LGM^93,96^, similar with the Pontic Mountains in NE-Turkey as shown by continuous glacier advances (Fig. 7d)^89^.

As mentioned above, clear evidences for palaeoenvironmental conditions during MIS-2 are missing in our loess record, but considering all mentioned palaeo-records, we prefer a scenario based on changed atmospheric circulation patterns to explain negligible loess formation during MIS-2 in NE-Armenia. Namely, we suggest an increase of westerly pathways crossing the Black Sea in an eastward direction being responsible for higher moisture availability in the Southern Caucasus area during MIS-2 along with more moderate temperatures. Via higher soil moisture and a consequently denser vegetation cover this moisture supply led to the stabilization of the former floodplain surface in the Kura Basin preventing large-scale deflation and therefore strong loess formation. Accordingly, still relatively elevated Black Sea SSTs (Fig. 7h)^94^ should have contributed to a higher moisture transfer to the eastern downwind areas, perhaps with the formation of a kind of "humid island" southeast of the Black Sea. Indications of ongoing soil formation in the LPS of NE-Armenia since late MIS-3 do likewise support this scenario, even though it is difficult to define the exact period of soil formation beyond doubt.

Another indication to the above scenario can be inferred from LPS located on the other side (in upwind areas) of the Black Sea as moisture source. E.g., LPS in the lower Danube Basin clearly lack evidence for higher humidity during MIS-2. Instead, they show maximum loess accumulation rates^25,66^. Regions north of the Greater Caucasus showed severe loess deposition during MIS-2, too^100,103^. In case of the lower Danube Basin, it is assumed that increased loess deposition during MIS-2 was caused by stronger effects of the SH that reached far west into E and SE Europe and blocked the Atlantic influences^25,66^. This, in turn, shows that the mountain range of the Greater Caucasus formed a kind of climate barrier that sheltered the Kura Basin and surrounding areas against the influences of the SH during MIS-2.

Summarizing, we demonstrate that loess deposition in NE-Armenia was seemingly linked to higher SH activity, while lacking or strongly reduced loess deposition during MIS-2 may show several reasons. However, based on supporting information from regional palaeo-records, we suggest landscape stabilisation due to higher moisture conditions that originated from the Black Sea.

## Methods

### Stratigraphic work and provenance survey

In total, we selected four profile sections for in-depths laboratory analysis. Overall, 328 samples were taken for soil physical, sedimentological and geochemical analyses. Sampling was generally oriented according to stratigraphic layers and soil horizons. For realizing the provenance survey, we took 33 samples from LPS BL (Supplementary Fig. S1), 24 samples from the Kura Basin (Supplementary Table S2), and 34 samples from the Southern Caucasus and Armenian Highlands (Supplementary Table S3).

Grain-size determination was conducted by pipette analyses and wet sieve techniques after dispersion with sodium pyrophosphate. Because the samples contained certain quantities of gypsum that disrupted the settling process in the sample cylinder by flocculation, all samples passed through a repeated cycle of dissolution and centrifugation until measured electrical conductivity fell below a value of 400 µS cm^−1^. The calculation of the GSI followed the procedure presented in^18^. The calcium carbonate content was determined by measuring the carbon dioxide gas volume after adding hydrochloric acid in a Scheibler apparatus. Soil organic matter was measured via suspension and catalytic oxidation (TOC-VCPN/DIN ISO 16904). Total iron content (Fe(t)-values) was determined after pressure digestion with concentrated nitric and hydrofluoric acid using atomic adsorption spectrometry. Pedogenic iron content (Fe(d)-values) was measured after dithionite extraction using atomic adsorption spectrometry as well.

The inorganic element concentration was determined using an Ametek X-ray Fluorescence Spectrometer^104^. The fine-grained fraction was extracted by sieving out material < 63 µm and drying it at 105 °C for 12 h. 8 g of sample material mixed with 2 g of Cereox analysis wax (Fluxana) were pressed to a pellet. Every sample was measured twice, rotating the pellet in between. Following the calculation procedure, root mean square errors of calibration and lower limit of detection can be inferred from SPECTRO (2007)^104^. Certified reference materials involving No. 5358-90, Nos. 2504-2506-83, Nos. 2507-2509-83, Nos. 2498-2500-83, NCS DC 73375, JF-1, UG-QLO- 1 and UG-SDC-1 were used for the calibration of the measurements. Element data were preprocessed by correcting for calcite, dolomite and gypsum according to^105^ and subsequent normalization of major element oxides based on the closure operation according to^106^.

All 328 samples as well as samples from the Kura Basin and the Armenian Highlands were used for rock magnetic measurements that were conducted at the Laboratory for Palaeo- and Environmental Magnetism at the University of Bayreuth. For this study, initial low field magnetic susceptibility was measured at frequencies of 0,3 and 3 kHz using the MAGNON VFSM susceptibility bridge (320 Am^-1^ AC field). This measurement characterizes the volume susceptibility К and divided by the density of the sample it delivers the mass specific susceptibility χ. For determination of the frequency dependent magnetic susceptibility (χ_fd_), we calculated the difference of mass specific susceptibilities measured at 0, 3 and 3 kHz, respectively. Moreover, the LPS were measured equidistantly every 5 cm using a Bartington MS2 susceptibility meter and S2F probe^107^ by taking the average of four measurements.

### Age determination

Luminescence ages were determined applying a post-IR-IRSL (pIRIR) protocol^108^ on polymineral fine grain (4–11 µm), using a first IRSL stimulation of 50 °C for 300 s, a pIRSL stimulation temperature of 225 °C for 300 s, and a preheat of 250 °C for 60 s. Typical pIRIR shine down curves and growth curves are shown in Supplementary Fig. S5a,b. The applicability of the protocol was confirmed by dose recovery tests and comparison with quartz OSL ages for the younger samples (< 60 ka)^39,109^. For older samples, especially within the MIS6 loess layer, ages were underestimated, probably due to fading. This was made evident by comparison with a tephra layer located in the lower part of the MIS6 loess, which was dated to 194.0 ± 8.5 ka^39^, and by laboratory fading tests^39,109^ Since laboratory fading rates often appear overestimated, measured fading rates were subtracted by a fixed rate of 1%, in order to represent the laboratory artefact responsible for the overestimation. This reduced fading rate was applied with the correction approach of^110^. Samples < 40 ka were not corrected for fading, because they showed fading rates < 1%. In total, at least seven aliquots were measured per sample, showing low standard deviations, as usual for fine grain aliquots with signals averaged over millions of grains. Radionuclide concentrations were determined either using a combination of alpha counting and ICP-OES, or a µdose system^111,112^. Dose rates were calculated with the program DRAC^113^.

## Supplementary Information

Supplementary Information.

## Data Availability

The authors declare that all data supporting this research are available within the paper, its Supplementary Information and Supplementary Data files.
