# Supplementary material for: Last glacial loess dynamics in the Southern Caucasus (NE-Armenia) and the phenomenon of missing loess deposition during MIS-2
Source: Sci Rep. 2022 Aug 2;12:13269. doi: 10.1038/s41598-022-17021-5 (PMC9345972; doi:10.1038/s41598-022-17021-5)
Supplement: Supplementary file 1 — Supplementary Information. [file 41598_2022_17021_MOESM1_ESM.pdf]

Supplementary Information

Last glacial loess dynamics in the Southern Caucasus (NE-Armenia) and the phenomenon of missing loess deposition during MIS-2

Daniel Wolf[1]*, Johanna Lomax[2], Lilit Sahakyan[3], Hayk Hovakimyan[3], Jörn Profe[2], Philipp Schulte[4], Hans von Suchodoletz[5], Christiane Richter[1], Ulrich Hambach[6], Markus Fuchs[2], Dominik Faust[1]

Affiliations:

[1]Institute of Geography, Technische Universität Dresden, Helmholtzstr. 10, 01069 Dresden, Germany.

[2]Department of Geography, Justus Liebig University Giessen, Senckenbergstr. 1, 35390 Giessen, Germany.

[3]Institute of Geological Sciences, National Academy of Sciences of the Republic of Armenia, Baghramyan Ave. 24a, 0019 Yerevan, Armenia.

[4]Department of Geography, RWTH Aachen University, Wüllnerstr. 5b, 52062 Aachen, Germany.

[5]Institute of Geography, Leipzig University, Johannisallee 19a, 04103 Leipzig, Germany.

[6]BayCEER & Chair of Geomorphology, University of Bayreuth, 95440 Bayreuth, Germany.

*Corresponding Author: daniel_wolf@tu-dresden.de (D.W.)

1. Stratigraphic information

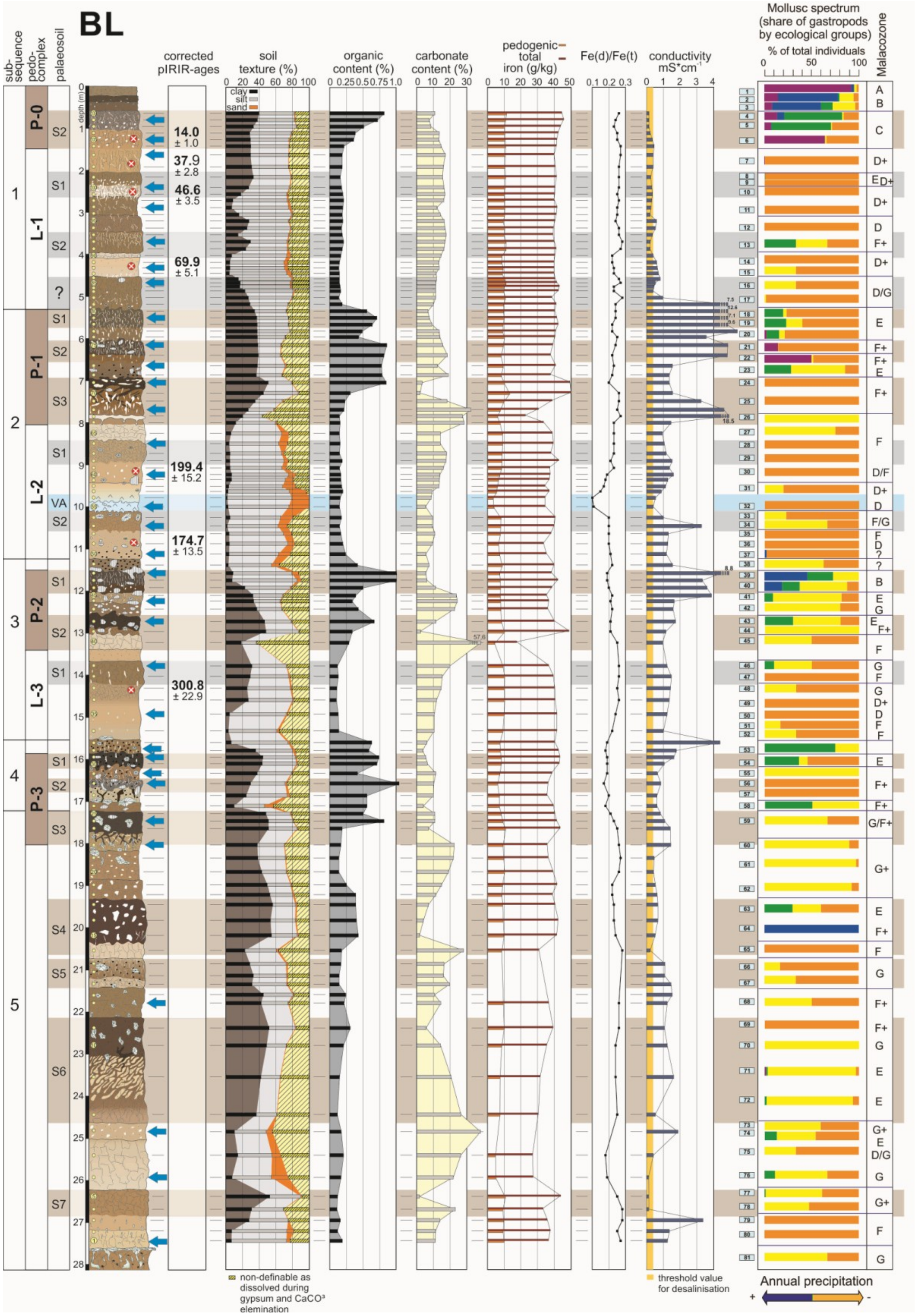

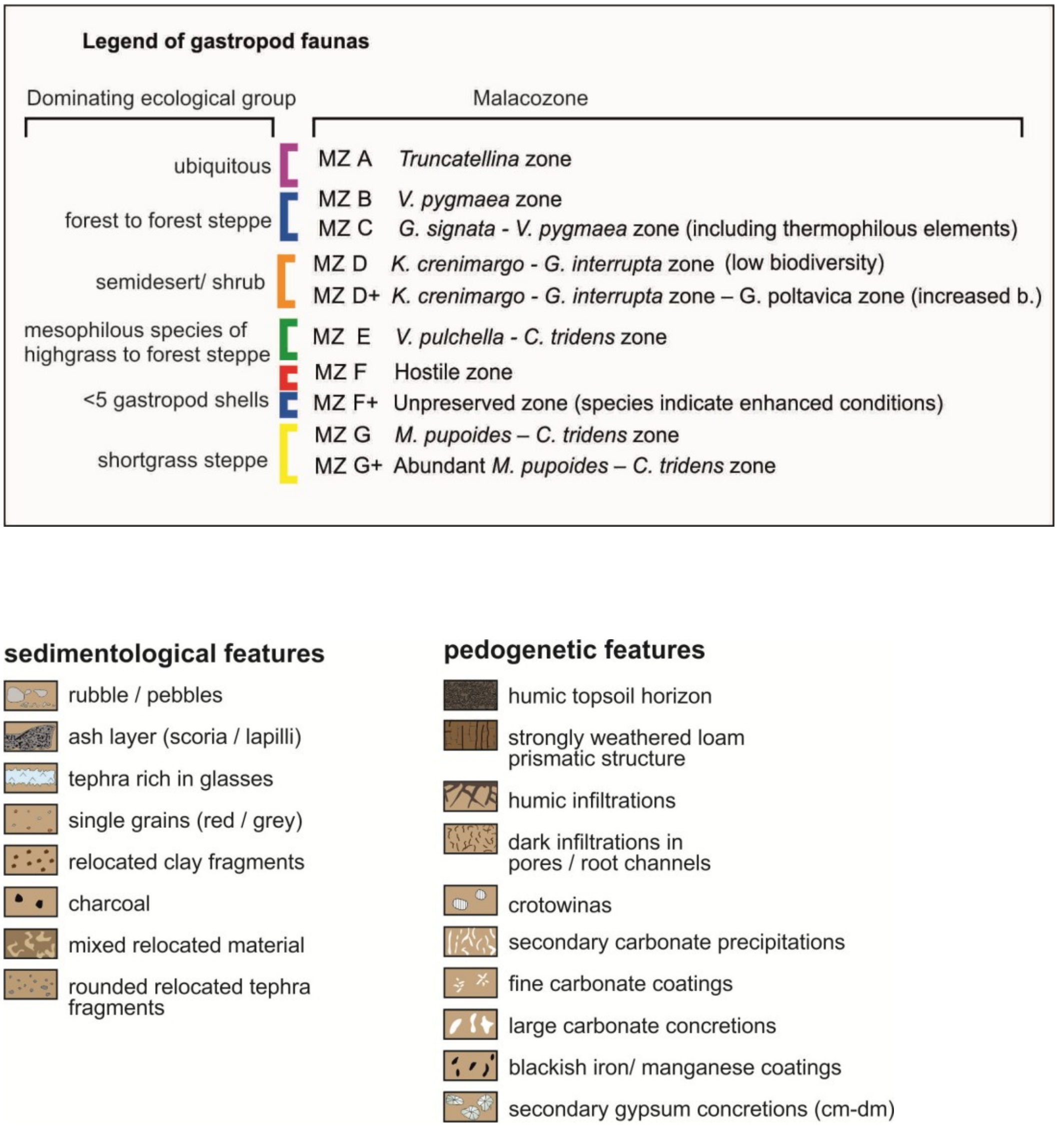

Supplementary Figure S1. Profile sketch of the LPS BL showing main pedostratigraphic units (sub-sequences, P - pedocomplexes, L - loess sequences, S - palaeosoils, VA - volcanic ash), fading corrected luminescence ages (post IR-IRSL 225), results of grain-size analyses, organic content, carbon content, iron activity, and electrical conductivity. On the right side, results of gastropod analyses are shown. Coloured bars show the allocation of each taxon to ecological categorisations; the arrow bar below visualises the implication on the associated moisture regime (see also Supplementary Figure S6). Blue arrows indicate samples used for XRF measurements.

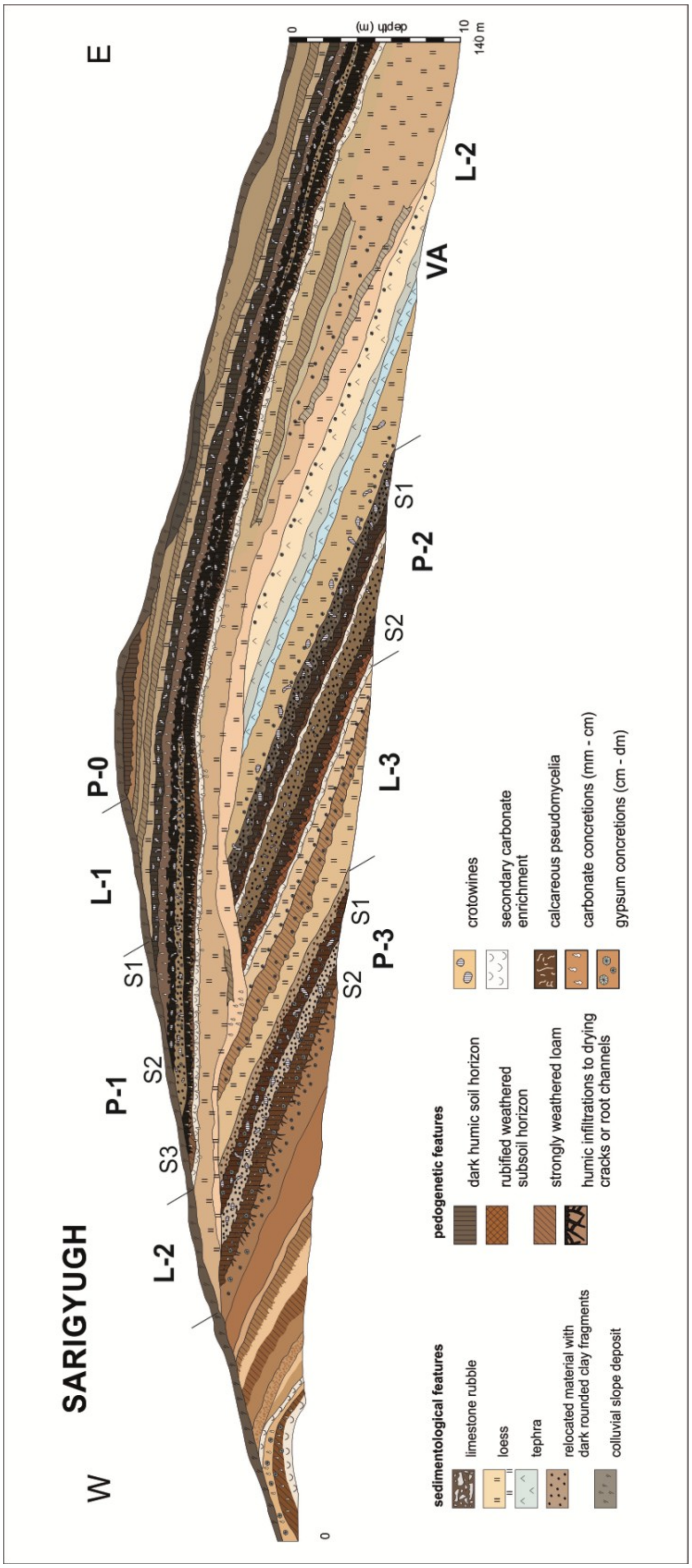

Supplementary Figure S2. Schematic cross-section of the LPS Sarigyugh road-cut with indication of main pedostratigraphic units. Note the strong erosion unconformity between the bluish volcanic ash (VA) and P-1 pedocomplex (Penultimate glacial).

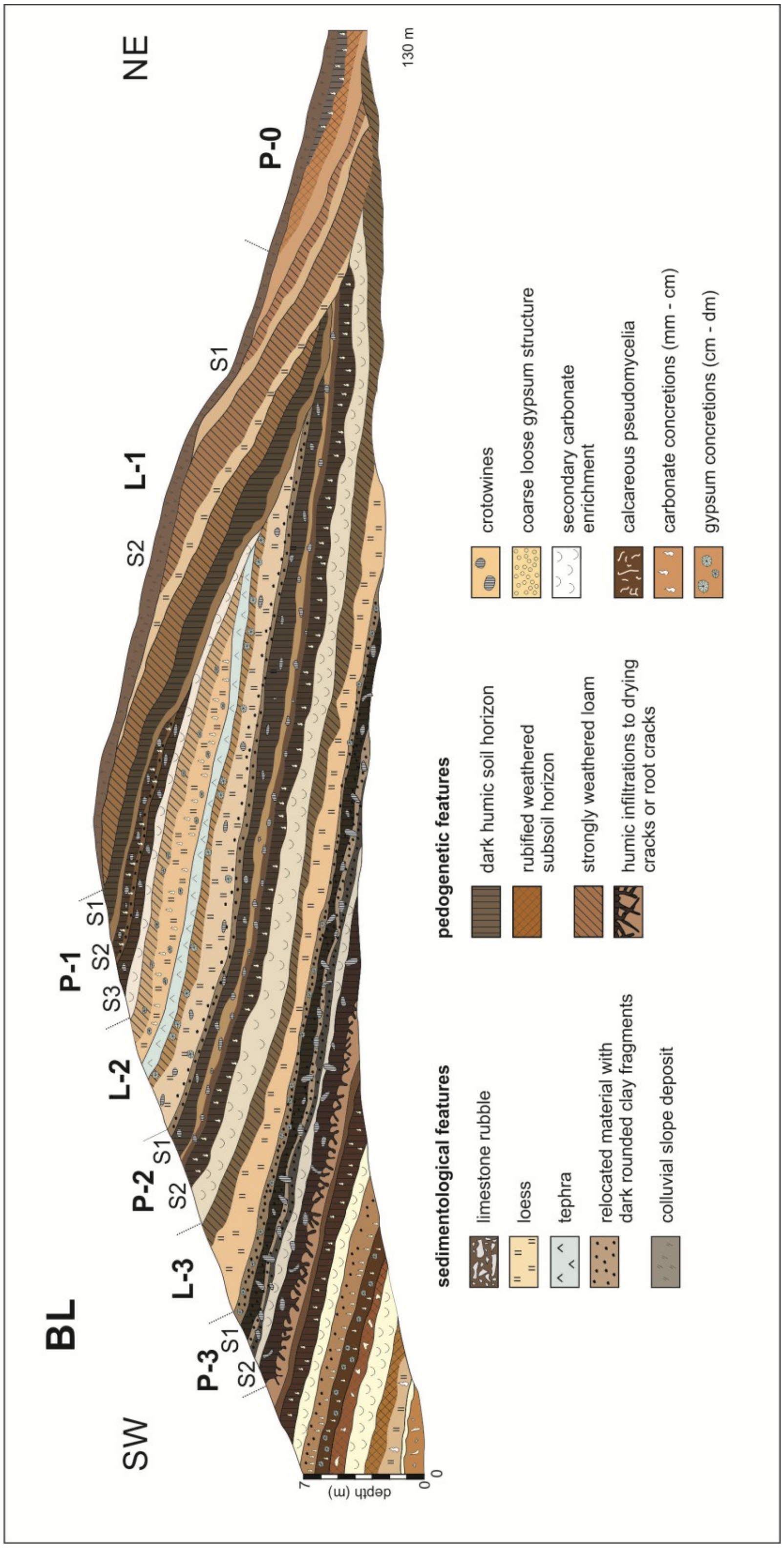

Supplementary Figure S3. Schematic cross-section of the LPS BL road-cut with indication of main pedostratigraphic units. Note the strong erosion unconformities within P-1 pedocomplex (MIS 5).

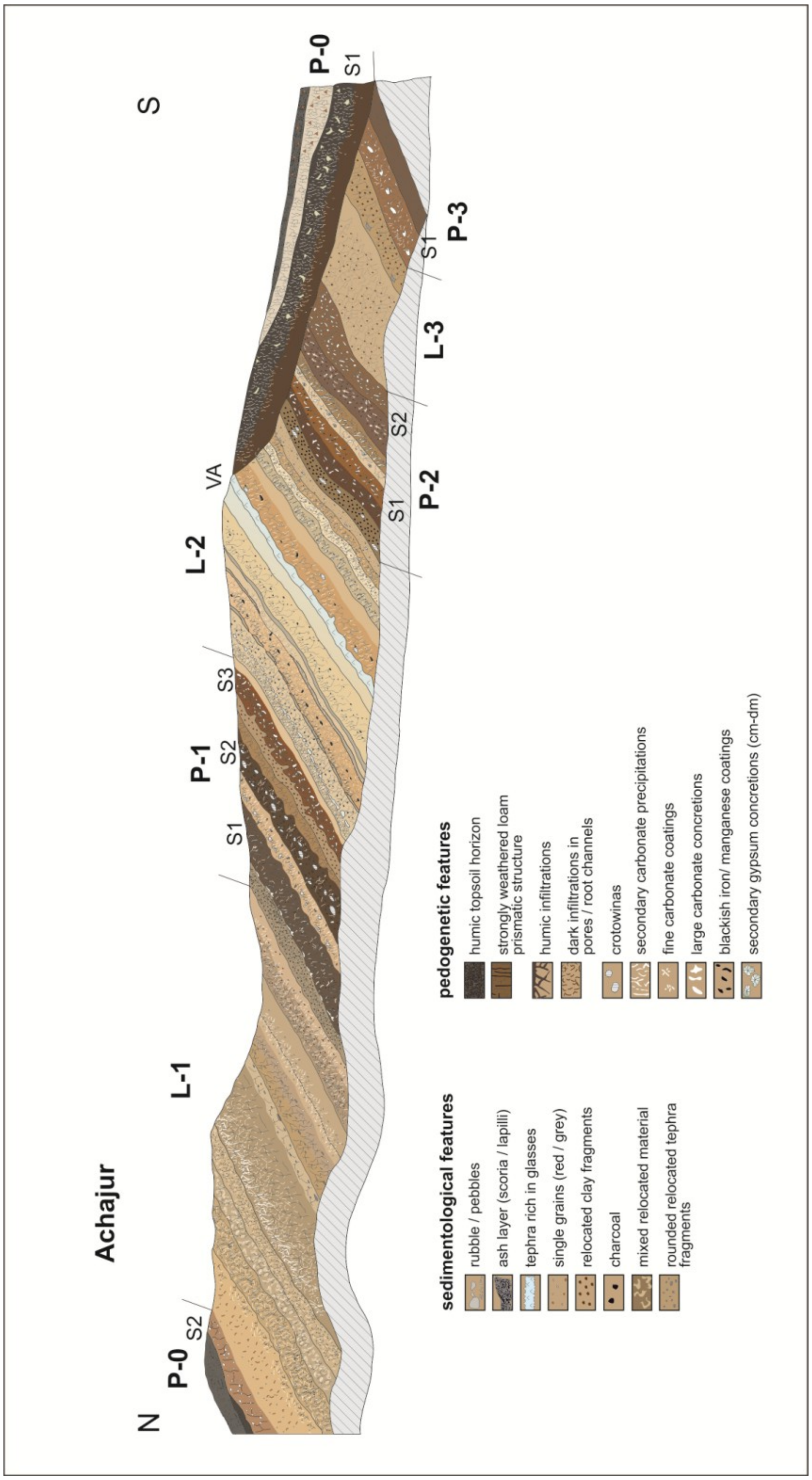

Supplementary Figure S4. **Schematic cross-section of the LPS Achajur** with indication of main pedostratigraphic units. The section is located in a saddle position and flanked by steep slopes. It thus reveals numerous relocation features like stone/ rock fragments, rounded soil and sediment fragments or relocated ash layers. Note the strong erosion unconformities within P-0 pedocomplex (Holocene period).

2. Luminescence Dating

Supplementary Table S1. Dose rate information and fading corrected pIRIR-225 D_e values and ages. Please note that ages for the Achajur section are non-corrected for fading in [1], and thus differ from ages provided here.

Section	Sample	Depth (m)	Dose rate (Gy/ka)	accepted aliquots for De determination	D_e (Gy)	Age (ka)
Sarigyugh	Gi621	0.5	3.19 ± 0.20	7	124.2 ± 6.3	38.9 ± 3.1
	Gi622	1.1	3.18 ± 0.20	10	159.4 ± 8.0	50.1 ± 4.1
	Gi624	2.9	3.84 ± 0.25	9	267.7 ± 13.4	69.7 ± 5.7
	Gi625	3.9	3.32 ± 0.22	10	276.1 ± 13.8	83.1 ± 6.8
	Gi626	4.9	2.70 ± 0.18	10	287.6 ± 14.4	106.4 ± 8.8
	Gi627	6.8	3.06 ± 0.20	10	507.1 ± 25.7	165.9 ± 13.7
	Gi628	8.3	3.10 ± 0.21	8	520.6 ± 26.3	168.2 ± 14.1
BL	Gi746	1.2	3.40 ± 0.18	8	47.5 ± 2.4	14.0 ± 1.0
	Gi747	1.8	3.18 ± 0.17	8	120.3 ± 6.0	37.9 ± 2.8
	Gi748	2.5	3.34 ± 0.19	8	155.6 ± 7.8	46.6 ± 3.5
	Gi749	4.3	3.75 ± 0.20	13	262.0 ± 13.2	69.9 ± 5.1
	Gi750	9.2	3.99 ± 0.22	6	795.6 ± 42.0	199.4 ± 15.2
	Gi751	10.7	3.88 ± 0.22	8	607.5 ± 31.0	174.7 ± 13.5
	Gi752	14.2	2.97 ± 0.17	7	770.5 ± 38.8	300.8 ± 22.9
Achajur	Gi538	2.1	3.00 ± 0.18	10	109.0 ± 5.5	36.3 ± 2.8
	Gi539	2.7	3.18 ± 0.19	10	119.1 ± 6.0	37.5 ± 2.9
	Gi540	3.5	3.15 ± 0.18	10	156.0 ± 7.8	49.5 ± 3.8
	Gi541	3.7	3.29 ± 0.19	10	164.4 ± 8.2	50.0 ± 3.8
	Gi542	5.5	3.17 ± 0.19	10	215.6 ± 10.8	68.1 ± 5.3
	Gi543	6.4	2.97 ± 0.18	10	212.0 ± 10.6	71.3 ± 5.5
	Gi544	7.0	3.00 ± 0.18	10	212.2 ± 10.6	70.7 ± 5.5
	Gi545	7.8	3.32 ± 0.19	10	237.8 ± 11.9	71.6 ± 5.5
	Gi546	8.5	3.37 ± 0.20	10	267.3 ± 13.4	79.4 ± 6.1
	Gi547	9.4	3.06 ± 0.18	10	275.9 ± 13.8	90.1 ± 7.0
	Gi548	9.7	3.20 ± 0.19	10	333.1 ± 16.7	104.0 ± 8.1
	Gi549	11.7	2.90 ± 0.17	10	410.7 ± 22.8	141.8 ± 11.6
	Gi550	12.3	3.33 ± 0.20	10	446.4 ± 22.6	134.1 ± 10.5

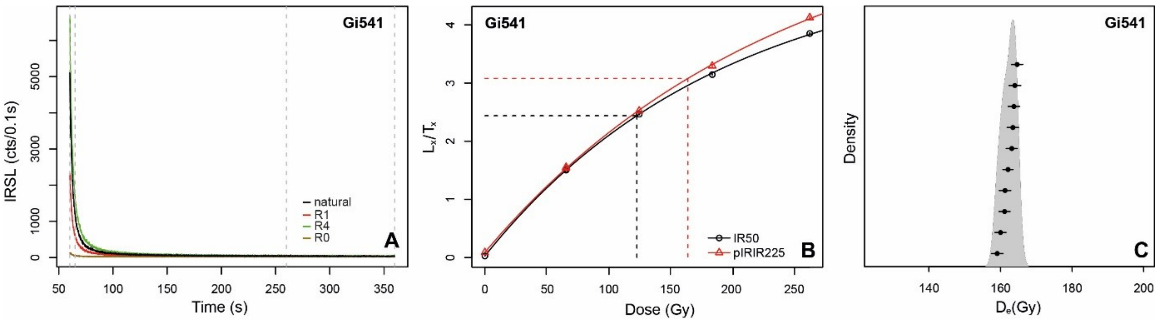

Supplementary Figure S5. (A) Shine down curve of one polymineral fine grain aliquot for the natural pIRIR signal, as well as laboratory generated signals. (B). IR50 and pIRIR-225 growth curves for the same aliquot shown in (A). (C) D_e and kernel density distribution of sample Gi541, indicating the low scatter of the polymineral fine grain measurements.

3. Geological information about the study area

The geological structure of the study area is characterized by Upper Cretaceous volcanic (basalts, basaltic andesites), volcaniclastic-sedimentary and sedimentary rocks. The Somkhet-Karabakh terrane (Fig. 2) belongs to the Eurasian active margin, had formed due to subduction of a northern branch of the Neotethys [2], and is interpreted as an island arc where Lower(?)-Middle Jurassic and Upper Jurassic [3] volcanic formations appear. Upper Jurassic and Lower Cretaceous volcanic rocks alternating with calcareous sedimentary and coarse volcaniclastic rocks cover the Middle Jurassic volcanic rocks [4]. Upper Cretaceous and Paleogene volcanic rocks unconformably cover the sequence of Jurassic to Lower Cretaceous volcanic and volcaniclastic rocks in parts of the Somkhet-Karabakh terrane [5]. The study area is dominated by Cretaceous limestone, sandstone and volcaniclastic sedimentary rocks. The Paleogene magmatic complex is post-collisional, which was active after the South-Armenian Microcontinent (SAM, Fig. 2) and the Eurasian active margin collision during the Late Cretaceous [2]. The Kura Basin itself is characterized by Miocene and Pliocene molasses as well as Pliocene and Quaternary sediments (Fig. 2) [2, 6].

4. Provenance Survey

Supplementary Table S2. Numbering, coordinates and depositional ages of samples from the Kura Basin.

**Sample List Provenance Analysis: Kura Basin**			
**Sample No.**	**Coordinates**	**Context**	**Sampled sections [7-9] + sampling depth**
**P-1**	N 41° 21' 04.3", E 45° 02' 50.6"	MIS 3 fluvial deposit	KH-7; 32m
**P-2**	N 41° 23' 25.6", E 44° 56' 27.8"	late MIS 2 fluvial deposit	KH-6; 5m
**P-3**	N 41° 21' 04.3", E 45° 02' 50.6"	MIS 3 fluvial deposit	KH-7; 6m
**P-4**	N 41° 21' 04.3", E 45° 02' 50.6"	MIS 3 fluvial deposit	KH-7; 14m
**P-5**	N 41° 21' 04.3", E 45° 02' 50.6"	MIS 5 fluvial deposit	KH-7; 34,5m
**P-6**	N 41° 24' 14.8", E 44° 50' 57.1"	late MIS 2 fluvial deposit	KH-4; 2m
**P-7**	N 41° 24' 14.8", E 44° 50' 57.1"	late MIS 2 fluvial deposit	KH-4; 5,5m
**P-8**	N 41° 24' 14.8", E 44° 50' 57.1"	MIS 3 fluvial deposit	KH-4; 14,5 m
**P-9**	N 41° 24' 14.8", E 44° 50' 57.1"	MIS 5 fluvial deposit	KH-4; 16,5m
**P-10**	N 41° 23' 25.6", E 44° 56'27.8"	late MIS 2 fluvial deposit	KH-6; 10m
**P-11**	N 41° 23' 28.3", E 44°49'27.1"	MIS 3 fluvial deposit	SH 7; 5m
**P-12**	N 41° 28' 21", E 44°42'12"	recent river bed-load	Khrami River at Nakhiduri
**P-13**	N 41° 21' 54", E 45° 04' 09"	>45 ka fluvial deposit	KU-1; 35m
**P-14**	N 41° 21' 54", E 45° 04' 09"	MIS 3 fluvial deposit	KU-1; 16m
**P-15**	N 41° 27' 23", E 45° 00' 34"	late MIS 2 fluvial deposit	KU-4; 3m
**P-16**	N 41° 27' 23", E 45° 00' 34"	MIS 2 fluvial deposit	KU-4; 20m
**P-17**	N 41° 23' 37", E 45° 07' 49"	early Holocene fluvial deposit	KU-6; 0,6m
**P-18**	N 41° 26' 09.1", E 44° 39' 05.2"	late MIS 2 fluvial deposit	MA-1; 1,2m
**P-19**	N 41° 26' 09.1", E 44° 39' 05.2"	MIS 3 fluvial deposit	MA-1; 3,2m
**P-20**	N 41° 35' 58", E 44° 57' 18"	recent river bed-load	Kura River at Rustavi
**P-21**	N 41° 19' 47", E 44° 50' 23"	recent river bed-load	Debeda River at Kvemo Sarali
**P-22**	N 41° 29' 45", E 44° 47' 26"	recent river bed-load	Algeti River at Marneuli
**P-23**	N 41° 26' 34", E 44° 40' 20"	recent river bed-load	Mashavera River at Khidiskuri
**P-24**	N 41° 23' 28.3", E 44° 49' 27.1"	MIS 3 fluvial deposit	SH 7; 3,5 m

Supplementary Table S3. Numbering, coordinates and depositional context of samples from the Southern Caucasus and Armenian Highlands.

**Sample List Provenance Analysis: Armenian Highlands**			
**Sample No.**	**Coordinates**	**Context**	**Photo of sampled section**
**P-25**	N 40° 28' 54.59", E 44° 46' 48"	alluvial fan with stone lines	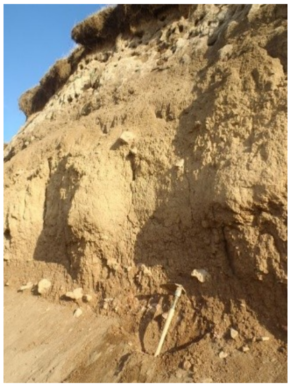
**P-26**	N 40° 37' 28.49", E 44° 56' 46.25"	thick alluvial fan with big gravel	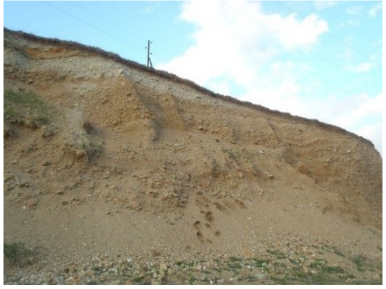
**P-27**	N 41° 0' 17.03", E 45° 9' 21.96"	weathered material from volcanic rock	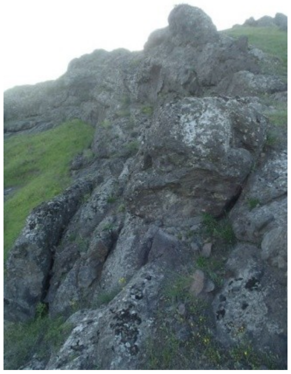
**P-28**	N 40° 54'48.99", E 45° 9' 26.71"	fluvial terrace with gravel/blocks; 15 m above recent river level	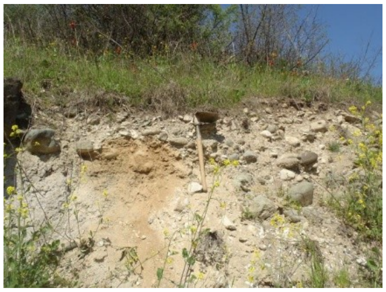

**P-29**	N 40° 55' 51.28", E 45° 9' 28.19"	upper part alluvial fan/ fluvial deposit; 8 m above recent river level	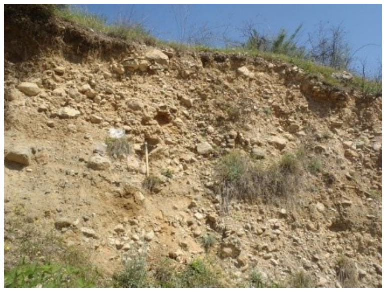
**P-30**	N 40° 55' 51.28", E 45° 9' 28.19"	lower part alluvial fan/ fluvial deposit; 8 m above recent river level	
**P-31**	N 40° 56' 4.92", E 45° 9' 44.42"	floodloam; terrace body; 5 m above recent river level	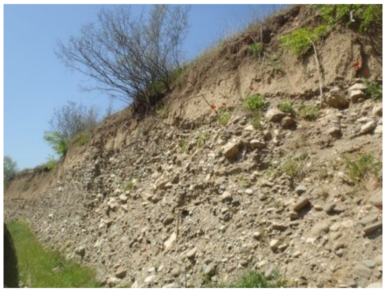
**P-32**	N 40° 56' 4.92", E 45° 9' 44.42"	gravel body; 5 m above recent river level	
**P-33**	N 40° 58' 8.4", E 45° 10' 35.00"	alluvial fan with angular rubble	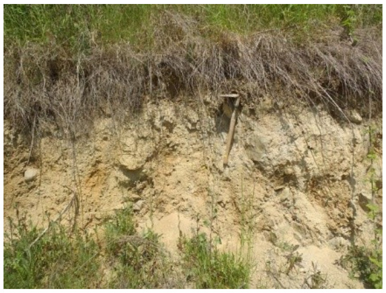
**P-34**	N 40° 58' 50.81", E 45° 11' 32.03"	fluvial/ alluvial deposit; angular/sub-angular; 20 m above recent river level	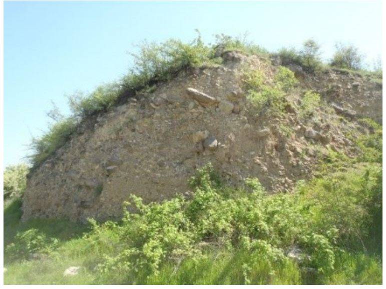
**P-35**	N 40° 59' 6.58", E 45° 11' 11.36"	alluvial fan, volcanic boulder-filled material	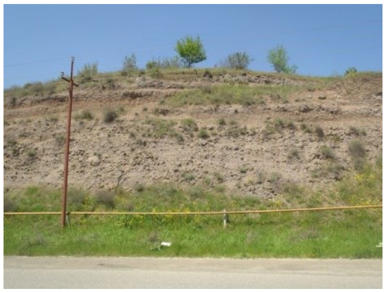

**P-36**	N 41° 0' 30.6", E 45° 10' 28.59"	rubble layer with angular calcareous rock material	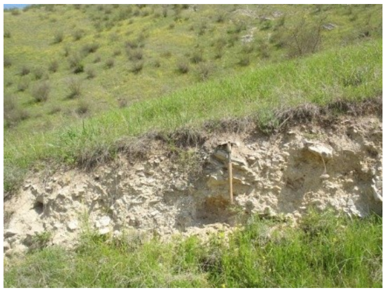
**P-37**	N 41° 2' 26.27", E 45° 6' 42.77"	alluvial fan, sub-angular stones	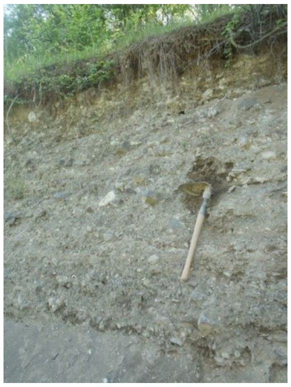
**P-38**	N 41° 2' 36.49", E 45° 6' 29.81"	min. 20 m thick alluvial fan, gravel/ boulders	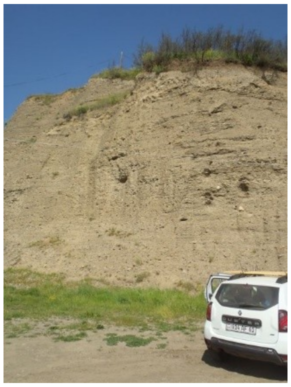
**P-39**	N 41° 4' 1.74", E 45° 4' 1.45"	slope debris, angular stones with up to 2m thick silt layers	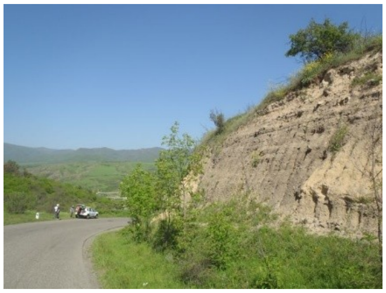
**P-40**	N 41° 4' 20.39", E 45° 4' 22.73"	fluvial terrace; 3 m above recent river level; rounded gravel	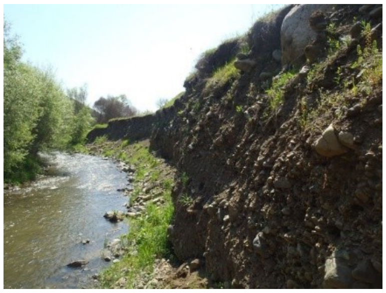

**P-41**	N 41° 4' 10.42", E 45° 3' 41.44"	fluvial terrace; rounded/sub-rounded gravel and loam (loess?), 10 m above recent river	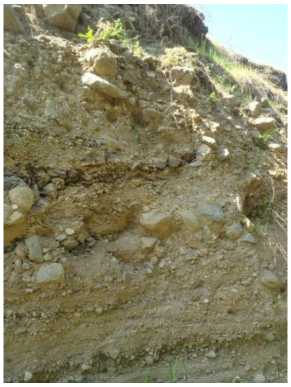
**P-42**	N 41° 4' 7.25", E 45° 3' 40.79"	big fan (20m) relocated silt (loess?) with gravel layers; 18 m above recent river level	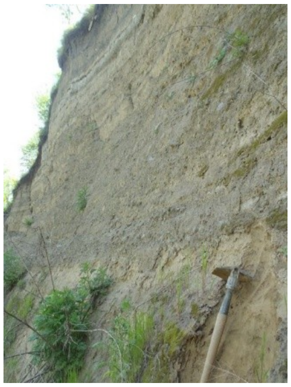
**P-43**	N 41° 4' 7.25", E 45° 3' 40.79"	base of big fan, gravel with volcanic sands; 4 m above recent river level	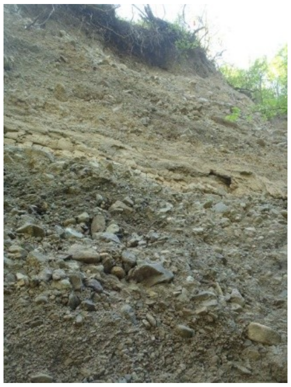
**P-44**	N 41° 4' 7.25", E 45° 3' 40.79"	base of big fan, gravel with brown loam matrix; 2 m above recent river level	
**P-45**	N 41° 4' 15.89", E 45° 4' 21.14"	sand lense from fluvial gravel body containing lots of fines; 20 m above recent river level	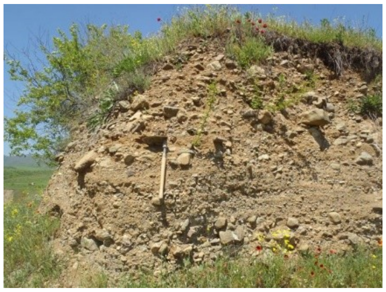

**P-46**	N 41° 4' 25.68", E 45° 4' 22.51"	alluvial fan; ~16 m thick; rounded gravel with silt layers; 40 m above recent river level	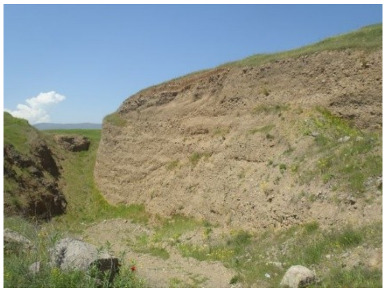
**P-47**	N 41° 3' 36.32", E 45° 3' 44.17"	weathered volcanic rock	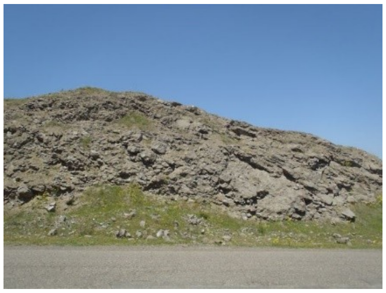
**P-48**	N 41° 2' 56.51", E 45° 4' 15.71"	weathered rock, clay/silt stone	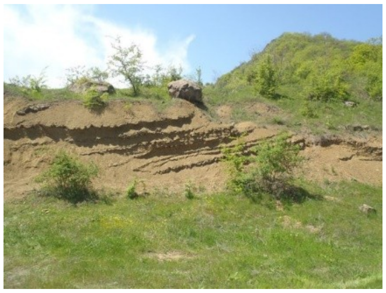
**P-49**	N 41° 2' 46.82", E 45° 6' 23.79"	fluvial gravel with large rounded boulders	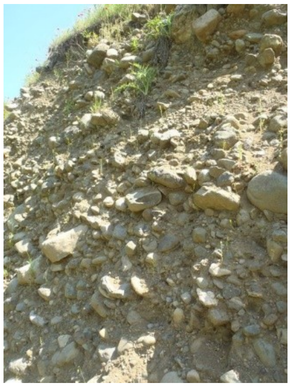
**P-50**	N 40° 51' 43.81", E 45° 7' 36.01"	alluvial fan with angular rubble and rounded boulders overlying limestone	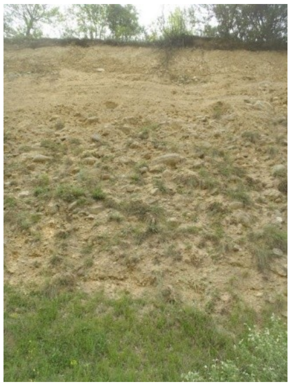

**P-51**	N 40° 49' 52.39", E 45° 6' 47.34"	alluvial fan with large rounded boulders over limestone	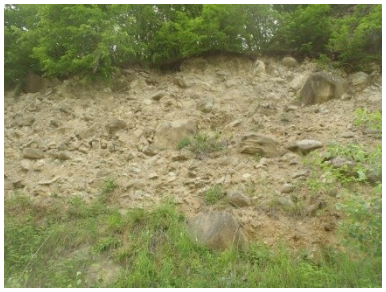
**P-52**	N 40° 37' 6.53", E 44° 57' 43.24"	fluvial gravel body close by Sevan Lake	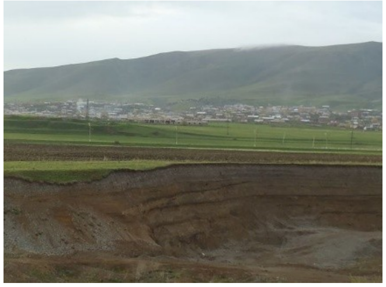
**P-53**	N 40° 36' 52.85", E 44° 57' 56.81"	3 m thick lake deposits, lower part	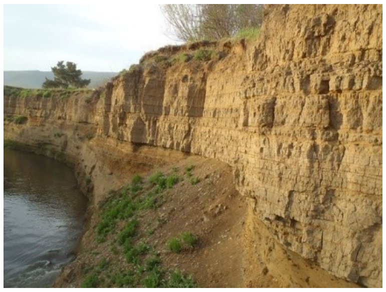
**P-54**	N 40° 36' 52.85", E 44° 57' 56.81"	3 m thick lake deposits, upper part	
**P-55**	N 40° 33' 8.06", E 44° 56' 30.52"	fine grained slope deposit	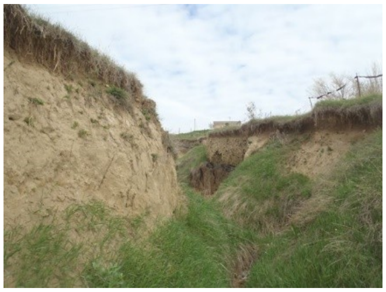
**P-56**	N 40° 33' 23.65", E 44° 54' 21.92"	Varser dust section, lower part	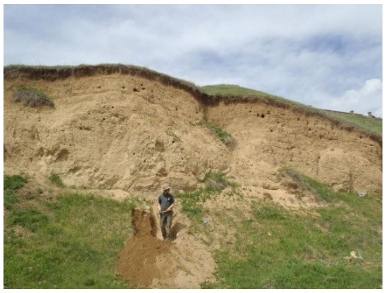

5. Palaeoenvironmental information provided by gastropod analyses

The mollusc diagram shows the fossil gastropod assemblages of BL. Significantly differing species compositions, biodiversity and abundance and peak phases of certain taxa allow a subdivision of the sequence into different malacozones. These show significant patterns in relation to the succession of pedocomplexes and loess, respectively [10]. Pedocomplexes predominantly include species of high-grass to forest-steppe biomes, indicating moister conditions for these sections. In contrast, loess layers which relate to glacial periods are associated with gastropod species of semidesert environments with shrub- and shortgrass-steppes, indicating semiarid to arid conditions. The upper section of the BL sequence, above 3 m depth, is dominated by typical semi-desert taxa such as *Imparietula* sp., *Pupilla poltavica*, *Pupilla kyrostriata*, *Gibbulinopsis interrupta* and *Kalitinaia crenimargo*. Between samples 7 and 6 (at 1.50 m depth) there is a significant shift in the gastropod composition. Here we have an abrupt disappearance of most semi-desert species and, in contrast, more moisture-demanding species such as *Vallonia pulchella, Vitrea pygmaea, Vallonia costata* & *Chondrula tridens* appear in the reddish subsoil of P-0. Conspicuous are the suddenly high abundances of these species, since environmental changes usually lead to a gradual increase in species that are better adapted to the new conditions. Such a sudden occurrence of high frequencies may indicate that the imbedding (soil)sediment has already been relocated. This would suggest a disconformity between mollusc samples 7 and 6, however, in the moment such disconformity is not supported by other analytical data. Similar disturbed depositional situations occur, e.g., between samples 12 and 11 or 22 and 21. The even stronger humic topsoil of P-0, including mollusc samples 5 and 4, shows an increase in moisture-demanding species that continue in the colluvial material, which, albeit, is dominated by ubiquitous species such as *Harmozica selecta*, *Truncatellina callicratis*, and *Truncatellina cylindrica*.

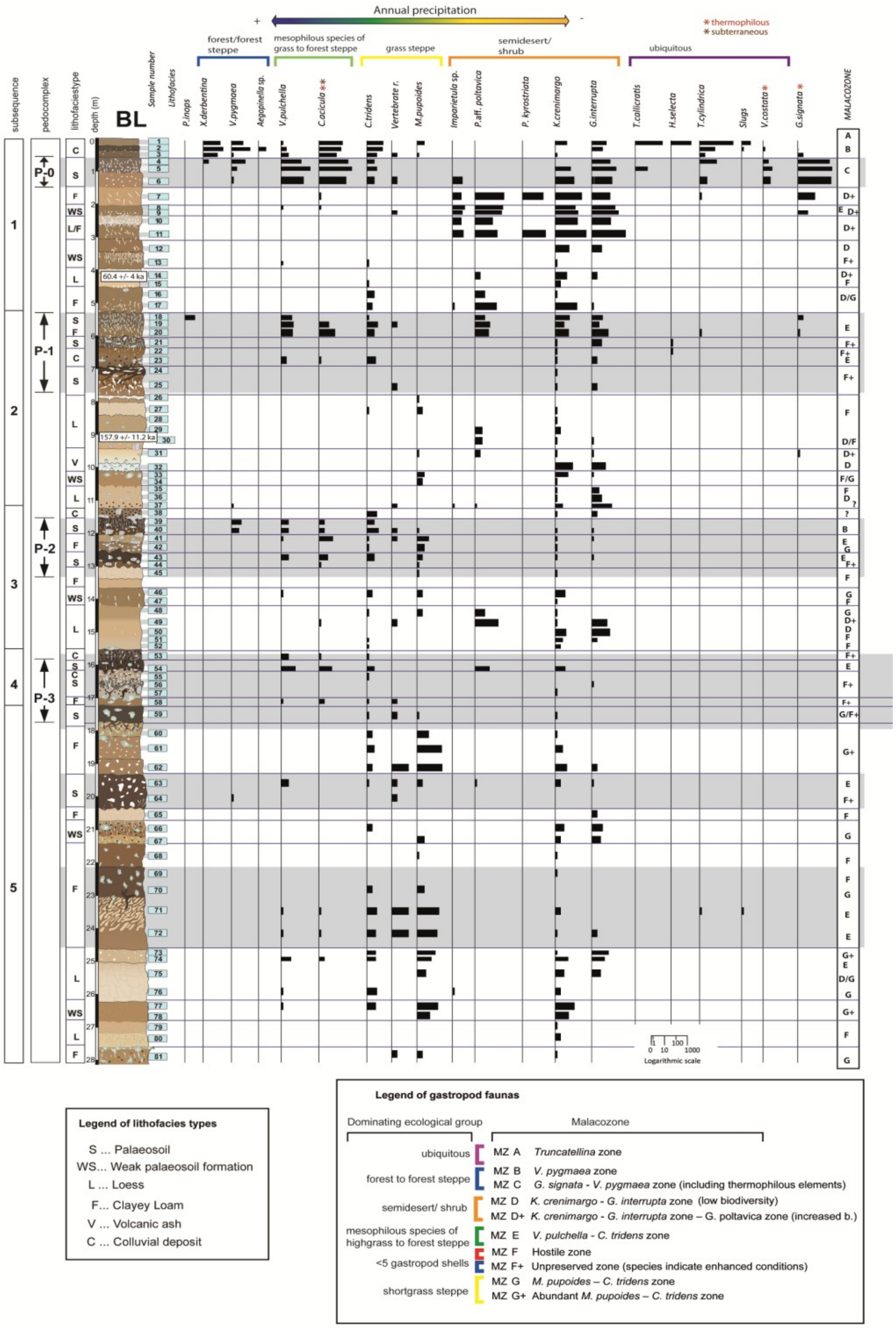

Supplementary Figure S6. Mollusc diagram illustrating the species composition and abundances for fossil gastropods of the BL section and biozonation based on the gastropod record. Coloured brackets at the top show the allocation of each taxon to ecological categorisations; the arrow bar visualises the implication on the associated moisture regime. Original data can be found [10].

References

1. Lomax, J. *et al.* Establishing a Luminescence-Based Chronostratigraphy for the Last Glacial-Interglacial Cycle of the Loess-Palaeosol Sequence Achajur (Armenia). *Front. Earth Sci.* **9**, 755084 (2021).
2. Sosson, M. *et al.* Subductions, obduction and collision in the Lesser Caucasus (Armenia, Azerbaijan, Georgia), new insights. . *Geol. Soc. Spec. Publ.* **340**, 329–352 (2010).
3. Galoyan, Gh. L. *et al.* To the petrology and geochemistry of Jurassic islandarc magmatics of the Karabagh segment of the Somkhet-Karabagh terrain. *Proceedings NAS RA, Earth Sciences* **66**, 3-22 (2013) (in Russian).
4. Kazmin, V. *et al.* Volcanic belts as markers of the Mesozoic–Cenozoic active margin of Eurasia. *Tectonophysics* **123**, 123–152 (1986).
5. Aslanyan, A.T. Regional Geology of Armenia. *Aipetrat, Yerevan* (1958) (in Russian).
6. Kharzyan, E. Geological Map of Republic of Armenia. *Ministry of Nature Protection of Republic of Armenia, Yerevan* (2005).
7. Suchodoletz, H. von *et al.* Late Pleistocene river migrations in response to thrust belt advance and sediment-flux steering e the Kura River (southern Caucasus). *Geomorphology* **266**, 53-65 (2016).
8. Suchodoletz, H. von, Gärtner, A., Zielhofer, C. & Faust, D. Eemian and post-Eemian fluvial dynamics in the Lesser Caucasus. *Quat. Sci. Rev.* **191**, 189– 203 (2018).
9. Suchodoletz, H. von & Faust, D. Late Quaternary fluvial dynamics and landscape evolution at the lower Shulaveris Ghele River (southern Caucasus). *Quat. Res.* **89**, 254-269 (2018).
10. Richter, C. *et al.* New Insights into Southern Caucasian Glacial-Interglacial Climate Conditions Inferred from Quaternary Gastropod Fauna. *J. Quat. Sci.* **35**, 634–649 (2020).
